# Structure elucidation and evaluation of the antimicrobial and antitumor activities of 5-methylthiazole-based Schiff base and its metal chelates

**DOI:** 10.1038/s41598-026-40320-0

**Published:** 2026-03-28

**Authors:** Khalid M. Wahdan, Hamada S. A. Mandour, Hoda A. El-Ghamry, Mohammed M. El-Gamil, Abdalla M. Khedr

**Affiliations:** 1 Chemistry Department, Faculty of Science, Tanta University, Tanta, Egypt; 2 Mansoura Laboratory, Department of Toxic and Narcotic Drug, Forensic Medicine, Medico Legal Organization, Ministry of Justice, Cairo, Egypt

**Keywords:** Schiff base, Thiazole ligand, Nanometric complexes, Spectral, Thermal, Antimicrobial, Antitumor, Cancer, Medical research, Chemistry

## Abstract

The current thiazole Schiff base ligand was obtained upon reacting 2-amino-5-methylthiazole with 2,4-dihydroxybenzaldehyde (**H**_**2**_**L**). The transition metal chelates then were presented by reacting the ligand with Mn(II), Cu(II), and Zr(IV) salts in 1:1 molar ratio forming chelates, **1**, **2** and **3**, respectively. The reaction of the ligand with Cd(CH₃COO)₂ afforded Cd(II) complex, **4**, in the molar ratio 2L:1 M. The type of contact that occurs between metal ions and the thiazole ligand was investigated by the assistance of magnetic susceptibility, FTIR, mass, UV–Visible and ^1^H NMR spectral and micro-elemental analyses. The resulting data demonstrated that the ligand–metal interaction happened via the phenolic O-atom *ortho*-position and the imine group N- atom which is dehydrogenated to form the metal complexes. Solvent molecules attached to the metal ions in complexes, whether present or absent were suggested using TGA in addition to thermodynamic activation parameters for the thermal decomposition steps were computed by the Eyring equation. Magnetic moment and UV–Visible measurements indicated formation of square planar Cu(II) chelate and octahedral Zr(IV) chelate, while the Mn(II) and Cd(II) ions formed tetrahedral metal chelates. XRD and TEM used to reveal structural microcrystalline data of both the organic compound (**H**_**2**_**L**) and inspected complexes. The patterns of X‐ray diffraction introduced the crystalline nature of each of the free ligand, complexes **1**, **3** and **4**, whereas complex **3** was relatively amorphous without an even dispersion of the solid constituents throughout the precipitation procedure. The study rigorously analyzed molecular structures using Density Functional Theory (DFT) calculations, revealing significant variations in ligand bond lengths and angles upon complexation, and demonstrating the electron-donating and accepting properties of HOMO and LUMO orbitals. Biological activity for the prepared compounds was accomplished. They exhibited high activity, especially after chelation. The Antimicrobial activity afforded very auspicious data upon comparison with the applied reference antibiotic. The greatest anti-cancer activity has been achieved by Cu(II)-complex **2** which showed IC_50_ value = 16.89 µg/ml against MCF-7 cell lines which is greater than the IC_50_ value of scaled drug applied (IC_50_ of 5-flurouracil = 28.0 µg/ml). Promising bioactive compounds’ binding affinities with HepG-2 and MCF-7 DNA helices were estimated to apply molecular docking.

**Supplementary Information:**

The online version contains supplementary material available at 10.1038/s41598-026-40320-0.

## Introduction

**Fig. 1:**
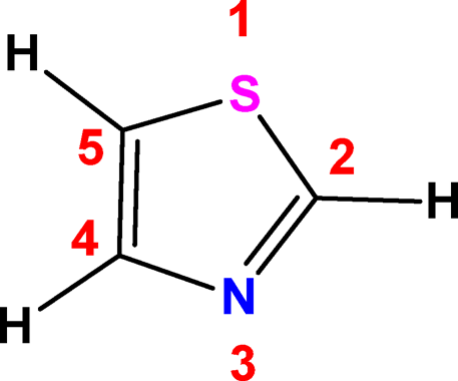
General structure of thiazole.

It is commonly recognized that nitrogen atoms are necessary for metal coordination at most biomolecules’ active locations. Heterocyclic compounds with nitrogen atoms in their molecular structure perform a stately task in discovery of novel pharmaceutical agents^1^. Azoles hold a special place in the fields of biology, chemistry, and medicine because they help create novel molecules with potential medical uses^2^. From azoles, we can point out thiazoles, which is considered as one of the most significant heterocyclic organic compounds, especially in the formulation of biomolecules displaying greater biological efficiency^3,4^. Thiazole and its derivatives display a broad range of properties, such as antimicrobial, antifungal, anticancer, antiviral, anticonvulsant, antidiabetic, anti-arthritic, and anti-inflammatory, anti-Parkinson, antiparasitic, antiulcer, analgesic and antithrombotic^5,6^. The formula of unsubstituted thiazole Fig. 1, C3H3NS, is flexible enough to enable new drug improvement. It is found in some natural compounds and in several synthetic substances^7^. As an example, vitamin B_1_ (thiamine) that contains the thiazole nucleus in its molecular structure plays the role of trapping electrons, contributes in the α-keto acids decarboxylation and facilitates the central nervous system’s (CNS) normal function by aiding in the production of acetylcholine^8^. Furthermore, thiazoles are a part of steroids, alkaloids and flavones^9^. The aromaticity of thiazole ring is promoted by the delocalization of one of the sulfur atom’s two electron pairs. Thiazole has also been greatly active owing to the acidity of the hydrogen on C2. Consequently, it possesses a crucial basic ability to produce new chemical entities^10^. Thiazole is capable of photochemical, dimerization, cycloaddition, arylation, oxidation, and substitution reactions. These modifications on the heterocycle ring resulted in numerous novel compounds with a variety of medicinal properties^11^.

Furthermore, many medications that are currently in use have a thiazole ring in their composition including Nitazoxanide (a wide-ranging antiparasitic and antiviral drug), Teneligliptin and Sodelglitazar ( used to in treatment of type II diabetes mellitus), Simeprevir and Faldaprevir (used to treat hepatitis C virus), Nizatidine and Famotidine (utilized to treat gastric reflux and peptic ulcers), Fanetizole, Sudoxicam, Fentiazac and Meloxicam (used as anti-inflammatory drugs) and Brecanavir, Ritonavir and Cobicistat (used in the treatment of HIV virus)^12,13^. Abafungin, Ethaboxam, Carbimazole, Isavuconazole, Ravuconazole, Mixothiazole and Thiabendazole (used as antifungal drugs)^14,15^. Additionally, there are numerous antibiotics with different structures, including penicillin and its derivatives (ampicillin and amoxicillin), sulfathiazole, tigemonam, pyrazmonam, aztreonam, carumonam, and others^16,17^. Aminothiazoles metal-based could replace the classic organic drugs. Their unique and powerful antibacterial, anticancer, antileukemic, antidiabetic, anti-inflammatory, and antiviral properties have been developed for use in medicine^18,19^. Their lipophilicity and ability to penetrate through the lipid membrane is an important characteristics that can be related with good biological activities^20^. Because of the high yield and increased bioactivities following chelation, the scientists are motivated to carry out this work in the future. In the continuation of our ongoing investigation into the beneficial role of chelation on molecules’ biological activity^21,22^, we right now record a new member of thiazole Schiff base ligands originated by the condensation reaction of 2-amino-5-methylthiazole with 2,4-dihydroxybenzaldehyde in addition to its transition metal complexes to investigate their anticancer, antifungal, and antibacterial qualities. DFT calculations and molecular docking studies will be studied.

## Experimental

The organic substances (2-amino-5-methylthiazole and β-resorcylaldehyde), transition metal salts (MnCl_2_.4H_2_O, CuCl_2_.2H_2_O, ZrCl_4_ and Cd(CH_3_COO)_2_) in high purity and delivered from Sigma-Aldrich.

### *Ligand (H*_*2*_*L) synthesis*

**Scheme 1:**
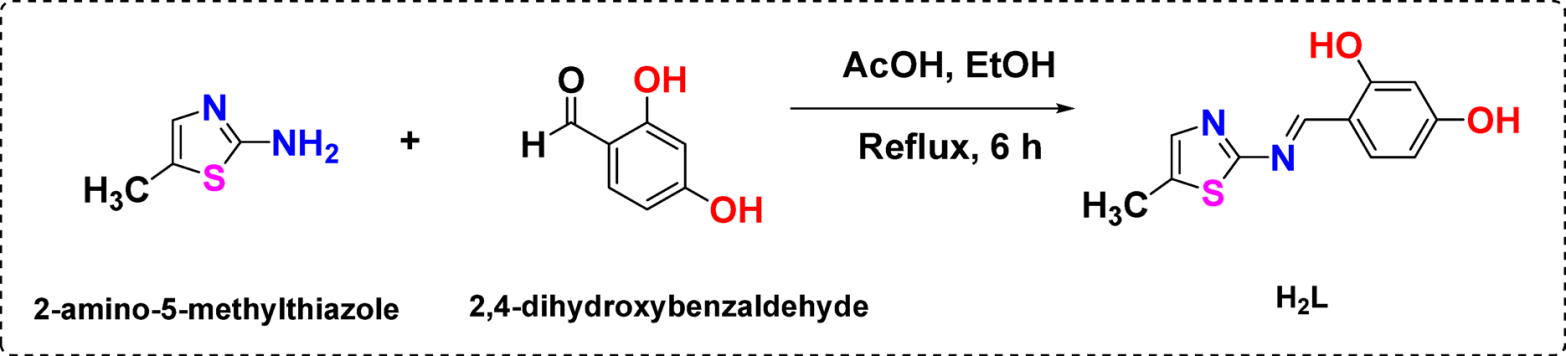
Ligand synthesis (**H**_**2**_**L**).

Preparation of Schiff base organic aminothiazole ligand was achieved by dropwise addition of 30 ml ethanolic solution (0.01 mol, 1.1417 gm) of 2-amino-5-methylthiazole to 30 mL of ethanolic solution (0.01 mol, 1.3812 gm) of β-resorcylaldehyde. A few drops of glacial acetic acid were then added to the reaction mixture. Then, six hours were spent with the liquid refluxing while being constantly stirred (Scheme 1). After then, the solution was allowed to cool to ambient temperature and then append to ice and refrigerate for two hours to finish the parent organic ligand’s precipitation. After filteration, the precipitate dried at room temperature and transformed into a yellowish powder. To obtain ligand purity, ethanol is utilized for recrystallization^23^.

### Synthesis of nanometric metal chelates

**Scheme 2:**
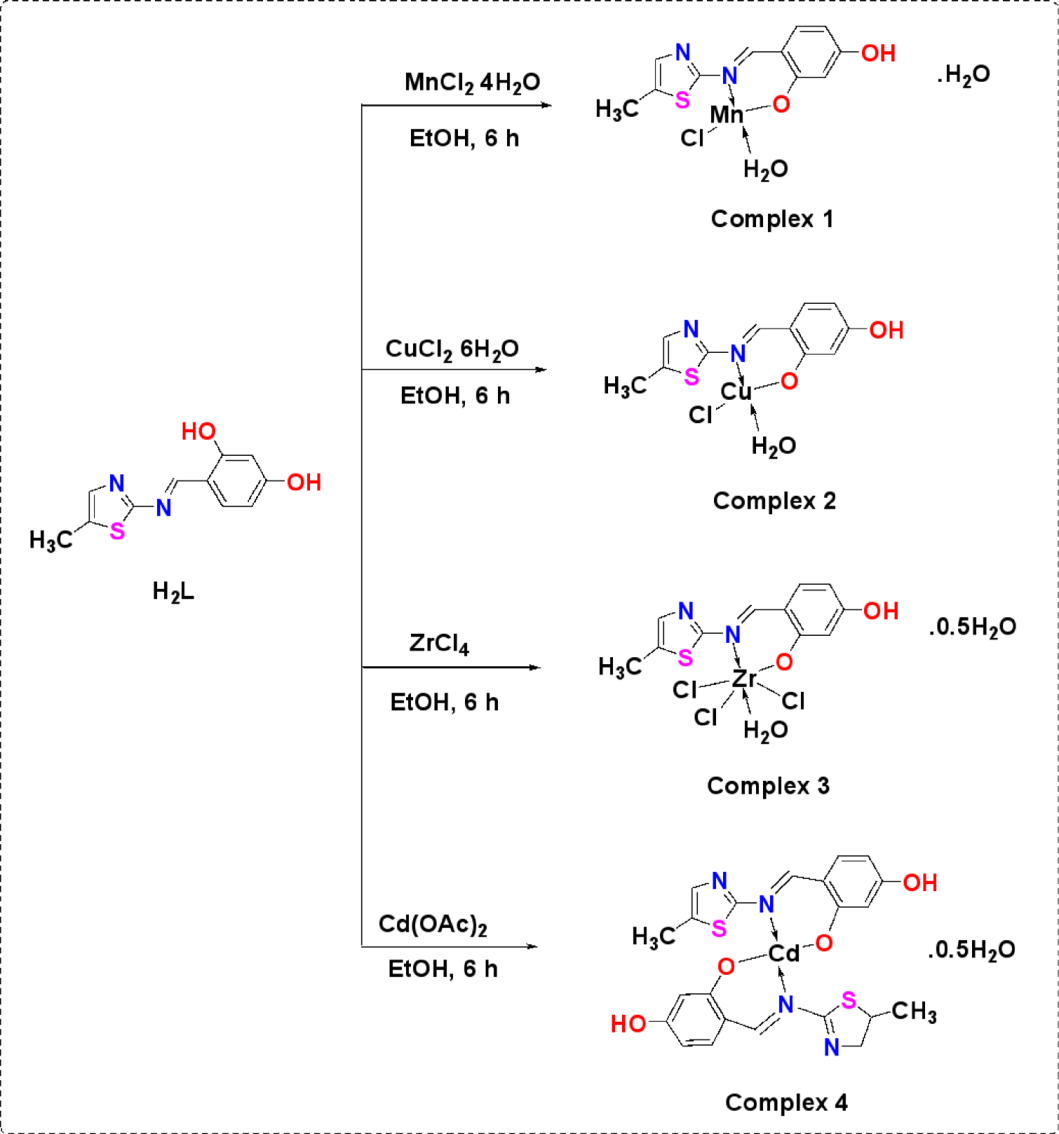
Nanometric metal complexes synthesis.

Thiazole Schiff base derivative (**H**_**2**_**L**) and transition metal salts MnCl_2_.4H_2_O, CuCl_2_.2H_2_O, ZrCl_4_ and Cd(CH_3_COO)_2_ was weighed in 1:1 molar ratio. Metal salt solution in 30 ml ethanol added to 30 ml of ligand-ethanolic solution. After two hours of refluxing the mixture, a few piperidine drops were added. The reaction is heated for three more hours before letting the temperature drop to room temperature. To obtain pure metal complexes, after filtering and washing the precipitated compound with ethanol, it was allowed to dry at ambient temperature (Scheme 2).

### Quantum chemical calculation

Quantum computations were conducted applying the DMOL^3^ program^24^, which is part of the comprehensive Materials Studio suite^25^. For the optimization of geometries, the RPBE^24^ functional was chosen due to its effective performance within the Generalized Gradient Approximation (GGA) exchange–correlation framework^26^. In addition, DFT (density functional theory) pseudopotential semi-core computations were employed alongside DNP basis sets^27,28^. This combination ensured high precision and reliability in the quantum mechanical computations, underscoring the rigorous approach of the research.

### Antibacterial and antifungal inspection

The Agar well diffusion method is used to screen the produced compounds for their antibacterial and antifungal properties. It is frequently used to evaluate the antibacterial qualities of microbial extracts or plants. A method such as the disk-diffusion approach is used to inoculate the surface of the agar plate by dispersing the volume of microbial inoculum throughout the entire surface. An aseptic perforation of a 6 to 8 mm diameter hole is then made using a sterile cork drill or bit. The well is then filled with 20–100 ml of the extract solution or antibacterial agent at the appropriate concentration. Subsequently, the agar plates are incubated according to the microorganism being tested. The antibacterial component diffuses throughout the agar medium and stops the tested microbial strain from proliferating^29^. The technique used mostly to test antimicrobial sensitivity is the well diffusion test. It provides both qualitative descriptive classification (e.g., susceptible or resistant) and quantitative data (zones of inhibition in millimeters). A well diffusion method for assessing mold susceptibility has been proposed by CLSI. A major advantage over the M38 is that results can be acquired utilizing this disk diffusion approach after 16 to 48 h of incubation^30,31^. Mueller–Hinton agar without supplements or a standard bacteriology lab Mueller–Hinton agar plates (pH 7.2–7.4 after gelling) are used in the CLSI disk technique because they provide convenient mold growth at 24 or 48 h. However, as some batches might not allow the appropriate development of organisms, each new batch of Mueller–Hinton medium should be evaluated for sufficiency using the CLSI criteria. As a result, the zones in the well (disk) diffusion test are typically larger than anticipated and may be above the allowed control limits^32^. After adjusting the inoculum suspension for the well method, agar plates must be infected within fifteen minutes, which follows the broth dilution standard protocol. The dried agar has three uniform streaks running over its whole surface. Once the agar surface has cured for at least 15 min, a sterile corn borer or tip was applied to aseptically punch a hole 6 to 8 mm in diameter. The well is then filled with 20–100 μL of the extract solution or antibacterial agent at the appropriate concentration. The plates should be incubated after the extract solution has been applied for fifteen minutes. If growth seems to slow down after 16 to 24 h (Mucoraceae), 2 h (A. fumigatus, A. flavus, A. niger), or 48 h (other species), the inhibition zone widths (in mm) surrounding the wells should be measured to the nearest full millimeter. If the plates do not grow sufficiently at the recommended intervals, they ought to be read later after being re-incubated^30–32^.

### Determination of antitumor activity (MTT protocol)

The studies were conducted at Cairo University’s microanalytical lab using human breast cells (MCF-7) and human liver cancer cells (HepG-2), which were provided by Vacsera (Giza, Egypt). A 96-well tissue culture plate was filled with 1 × 10^5^ cells/ml (100 µl/well) and cultured at 37 °C for 24 h to produce a complete monolayer sheet. When a cell confluent sheet had developed, growth medium was removed from 96-well microtiter plates, and the cell monolayer was removed twice using wash media. In RPMI medium (maintenance medium) with 2% serum, the tested substance was diluted twice. After 0.1 ml of each dilution was tested in individual wells, producing just maintenance medium, three wells were used as a standard control. The plate was incubated at 37 °C before being examined. Physical indicators of toxicity, such as cell granulation, rounding, shrinkage, and partial or whole loss of the monolayer, were examined. Each well received twenty microliters of a 5 mg/ml MTT solution in PBS (BIO BASIC CANADA INC.). To completely incorporate the MTT into the medium, spin it at 150 rpm for five minutes on a shaking table. To allow the MTT to digest, incubate at 37 °C with 5% CO_2_ for four hours. After emptying the medium, wipe away any remaining residue with paper towels if needed. The MTT metabolic product Formazan needs to be reconstructed using 200 µl of DMSO. Place the formazan on a shaking table and spin it at 150 rpm for five minutes to fully combine it with the solvent. After subtracting the background at 620 nm, measure the optical density at 560 nm. There should be a direct correlation between optical density and cell count^33,34^.

### Molecular docking

Molecular docking simulations are crucial for scientists around drug research and production, as they help propose models of drug interactions and provide insights into the behavior of new drugs towards biological targets. These theoretical calculations play a pivotal role in understanding how potential drugs might interact with their targets at the molecular level, which is necessary for the rational creation of an effective treatment.

#### Preparation of Ligands

The target compounds, optimized for docking, were meticulously prepared applying the Schrödinger suite’s LigPrep program,^35^ following its default methodology. To accurately model the metal centers within the complexes, the OPLS-2005 force field was employed to assign partial charges and steric parameters. The metal complexes geometries treated as rigid bodies during the docking process to preserve the coordination sphere**.** This preparation process ensures that the ligands are in their optimal conformations for docking studies, which is critical for accurate and reliable results.

#### Protein preparation

HepG-2 (PDB ID: 2W3L) and MCF-7 (PDB ID: 3W2S) three-dimensional structures were obtained from the Protein Data Bank^36,37^. The proteins have been prepared applying the Schrödinger suite’s Protein Preparation Wizard^35^, which removed H_2_O molecules within a 5 Å radius, eliminated small molecules, established disulfide bonds, and added hydrogen atoms. Constrained minimization was performed with default parameters using the OPLS-2005 force field. The inner box is centered on the co-crystal ligand, while the outer box size is determined by the native ligand size using the Glide grid generating panel (dimensions: 13 Å × 13 Å × 11 Å for 2W3L; 16 Å × 12 Å × 16 Å for 3W2S). The metal ions present in the receptor structure were treated as part of the rigid protein frame, with charges and Van der Waals parameters defined by the OPLS-2005 force field to ensure accurate electrostatic mapping.

These meticulously prepared protein structures ensure that the docking simulations are conducted on accurate and representative models of biological targets. Docking studies were conducted using the Glide program^35^ in Schrödinger’s suite. The Glide Dock XP procedure was utilized to dock every drug under investigation to the target protein. The G-score indicates how well a compound binds to a receptor, while the RMSD value compares the binding conformation in relation toward a reference for a binding configuration. Potential inhibitors can be identified using both G-score and RMSD values^38^.

## Results and discussion

### Microanalysis and conductance inspections

Although the complexes being studied can completely dissolve in DMSO and DMF, they dissolve extremely poorly in most common organic solvents. The results of elemental microanalysis of the thiazole derivative, **H**_**2**_**L**, and formed nano-sized complexes (**1**–**4**) are surprisingly in accordance with their respective chemical formulas. According to the results in Table 1, the Mn(II), Cu(II), and Zr(IV) complexes formed with a 1:1 (M:L) stoichiometric ratio, while the Cd(II) complex formed with a 1:2 (M:L) ratio. At a 1 × 10^–3^ M nanosized complex solution in DMF, the molar conductance values acquired for complexes **1**─**4** discovered to fall between 8.15 and 11.55 Ω^−1^ cm^2^ mol^−1^, supporting that all of them exhibit non-electrolytic character. The complexes under study are not hygroscopic which concluded upon exposure of them to open air^39^.

**Table 1:** Microanalytical and physical information about the thiazole derivative **H**_**2**_**L** and complexes 1–4.

Comp	Molecular formula (Empirical formulae)	M. wt	Yield (%) (M.P.°C)	Color (Λm)	Elemental Analysis Found (Calc.)			
					%C	%H	%N	%M
Lig	H_2_L C_11_H_10_N_2_O_2_S	234.27	73.25 (185)	Yellow	56.49 (56.40)	4.41 (4.30)	12.19 (11.96)	–
1	MnC_11_H_13_N_2_O_4_SCl [Mn(HL)Cl(H_2_O)]·H_2_O	359.69	78.13 (310)	Dark red (9.76)	36.24 (36.73)	3.91 (3.64)	7.53 (7.79)	15.56 (15.27)
2	CuC_11_H_11_N_2_O_3_SCl [Cu(HL)Cl(H_2_O)]	350.28	70.02 (325)	Dark green (8.15)	37.52 (37.72)	3.42 (3.17)	8.33 (8.00)	18.83 (18.14)
3	ZrC_11_H_12_N_2_O_3.5_SCl_3_ [Zr(HL)Cl_3_(H_2_O)]·0.5H_2_O	457.87	60.14 (315)	Red (11.55)	28.53 (28.85)	2.85 (2.64)	6.21 (6.12)	19.32 (19.92)
4	CdC_22_H_19_N_4_O_4.5_S_2_ [Cd(HL)_2_]·0.5H_2_O	587.95	57.01 (325)	Brown (9.56)	44.53 (44.94)	3.38 (3.26)	9.31 (9.53)	19.87 (19.12)

### FT-IR spectroscopy

**Fig. 2:**
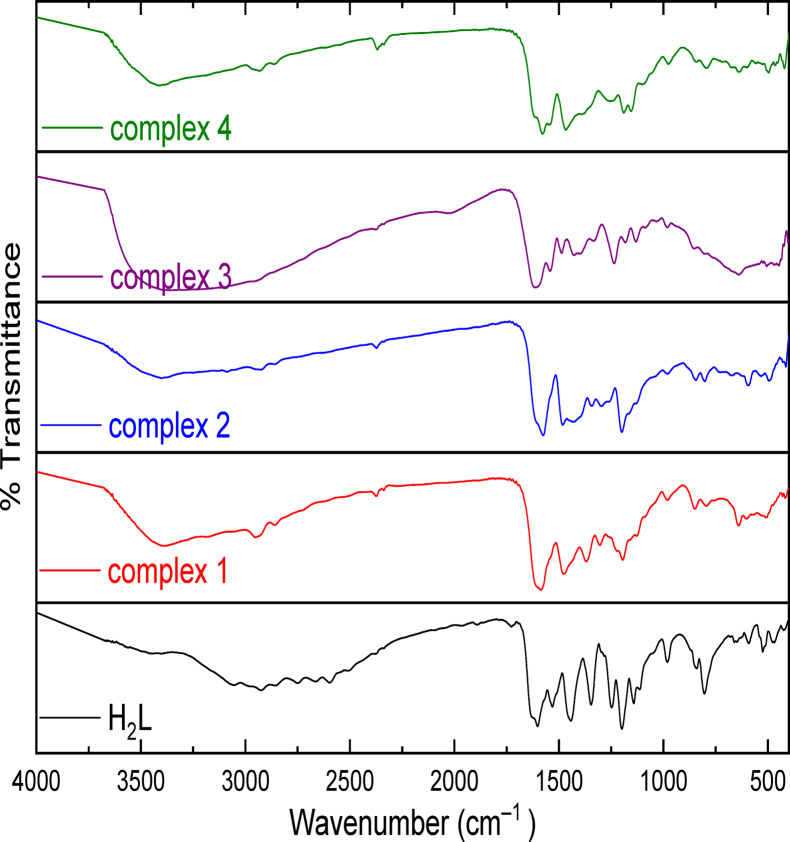
Mn(II), Cu(II), Zr(IV), and Cd(II) FT-IR spectra of chelates with the thiazole ligand **H**_**2**_**L.**

Infrared spectroscopy is a powerful method which can be used in characterization of newly prepared compounds^40–42^. The main vibrational bands of the spectrum of the aminothiazole ligand (**H**_**2**_**L**) and the metal complexes **1**–**4,** Table 2 displays them together with their assignments. Modes of which the ligand **H**_**2**_**L** can bind with the metallic ion sites incorporated in the complexes were determined by simple comparison of thiazole derivative, **H**_**2**_**L,** spectrum with the acquired nanometric complexes (Fig. 2). Based on this analogy, the ligand spectrum exposed the presaged bands of υ(OH), υ(C = N) imine, υ(C = N) ring and υ(C–O) at 3445, 1630, 1532 and 1247 cm^-1^ respectively. Apart from the band corresponding to C = N of thiazole ring which appeared at 1531 cm^-1^ in spectra of complex **2** and at the same ligand position in spectra of the rest of complexes, the other bands clearly shifted in their positions upon the formation of complexes. We can realize the imine band that appeared in **H**_**2**_**L** spectrum at 1630 cm^-1^, downfield shifted in complexes’ spectra and appeared in the range 1574–1591 cm^-1^. Also, the stretching vibration band for C-O bond appeared in the complexes’ spectra in the range 1223–1258, affording a shift of 11–24 cm^-1^ compared with its position in the ligand spectrum: at 1247 cm^-1^. This demonstrates how the imine nitrogen atom and dehydrogenated oxygen contribute to the ligand’s ability to chelate to the metal centers. This is also backed by the new bands that appeared in the complexes at 594–641 cm^-1^ and at 413–422 cm^-1^ attributable to M– O and M– N bonds respectively^43,44^.

**Table 2:** Identification of vibrational spectral bands in **H**_**2**_**L** and the checked nanosized chelates FT-IR spectra with major importance.

CPD	υ(OH, H_2_O)	υ(C = N) _imine_	υ(C = N) _ring_	υ(C-O)	υ(M–O)	υ(M–N)
Ligand	3445, 3392	1630	1532	1247	–	–
1	3394	1583	1532	1223	506	460
2	3400	1574	1531	1258	534	486
3	3392	1591	1532	1235	504	435
4	3410	1579	1532	1235	506	452

### ^*1*^*H NMR spectra*

The proton nuclear magnetic resonance ^1^H NMR technique is a spectroscopic method for observing the local magnetic fields surrounding the nuclei of hydrogen atoms. It is classified as one of the most useful ways for verifying binding styles of complexation. ^1^H NMR spectra are expressed in (Figs. S1–S3 and Table S1). In the free ligand ^1^H NMR spectrum, several major peaks were agreed upon when the ligand structure was provided. The ligand spectrum exhibited the two protons of -OH groups as singlet peaks at 12.02 and 10.54 ppm. The Cd(II) and Zr(IV) complexes’ spectra showed that the initial signal vanished, indicating that the complexes were generated by deprotonating the *ortho*-position OH group. The ligand band appearing at δ = 9.023 ppm, assignable to azomethine protons, was shifted in the complexes’ spectra and appeared at δ = 8.93 and 9.36 ppm in Zr(IV) and Cd(II) complexes, respectively, confirming the azomethine nitrogen’s participation in complex formation. Additionally, the ligand spectrum showed signals at 6.34, 6.45, and 7.58 ppm due to the phenyl protons. The thiazole proton was identified as the singlet that showed at 7.33 ppm, whereas the methyl group protons were identified at 2.42 ppm. The combination of the results obtained from FT-IR spectra was confirmed by ^1^H NMR data, which assured that the free ligand chelates with the metallic ions through the imine group’s nitrogen atom and the phenolic group’s oxygen atom in the ortho-position, following deprotonation^45–47^.

### Mass spectral-analysis

**Fig. 3:**
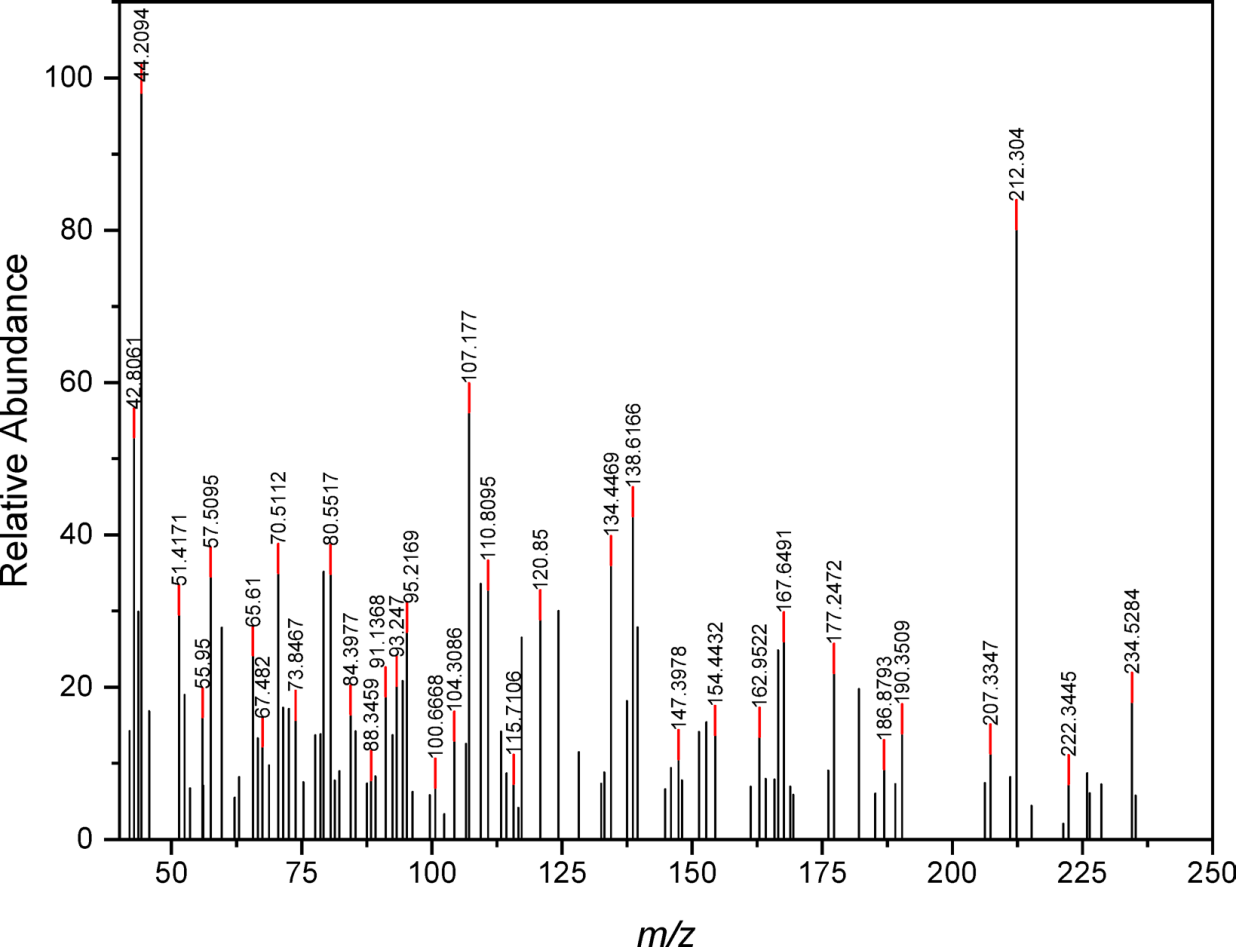
Mass spectrum of thiazole Schiff base ligand **H**_**2**_**L**.

The four prepared metal chelates (**1**–**4**) together with the free ligand (**H**_**2**_**L**) have been inspected using mass spectrometry over m/z = 40–1000 range at 70 eV. This provides enough evidence to clarify the molecular weights and, consequently, the molecular formulas of the newly synthesized materials^48,49^. Mass spectra of thiazole derivative **H**_**2**_**L** and the acquired nanosized chelates **1**–**4** are presented in Fig. 3 and Figs. S4–S7. The molecular ion peaks in these figures can be seen at m/z = 234.5, 358.2, 350.01, 456.7, and 588.12 for **H**_**2**_**L** and complexes **1**–**4**, respectively, which are compatible with the computed molecular weights of 234.27, 359.69, 350.28, 457.87, and 587.95, and hence confirm the predicted molecular weights of the ligand and complexes. The base peaks of **H**_**2**_**L** and complexes **1**–**4** appeared at m/z = 44.20, 140.09, 188.23, 100.13, and 102.19, assigned to the fragments C_3_H_6_, C_5_H_4_N_2_OS, C_4_H_4_CuN_2_OS, C_4_H_5_NS, and C_4_H_6_NS, respectively. Schemes S1-S5 represent the proposed mass fragmentation patterns of the substances under inspection.

### Electronic spectra and magnetic properties

**Fig. 4:**
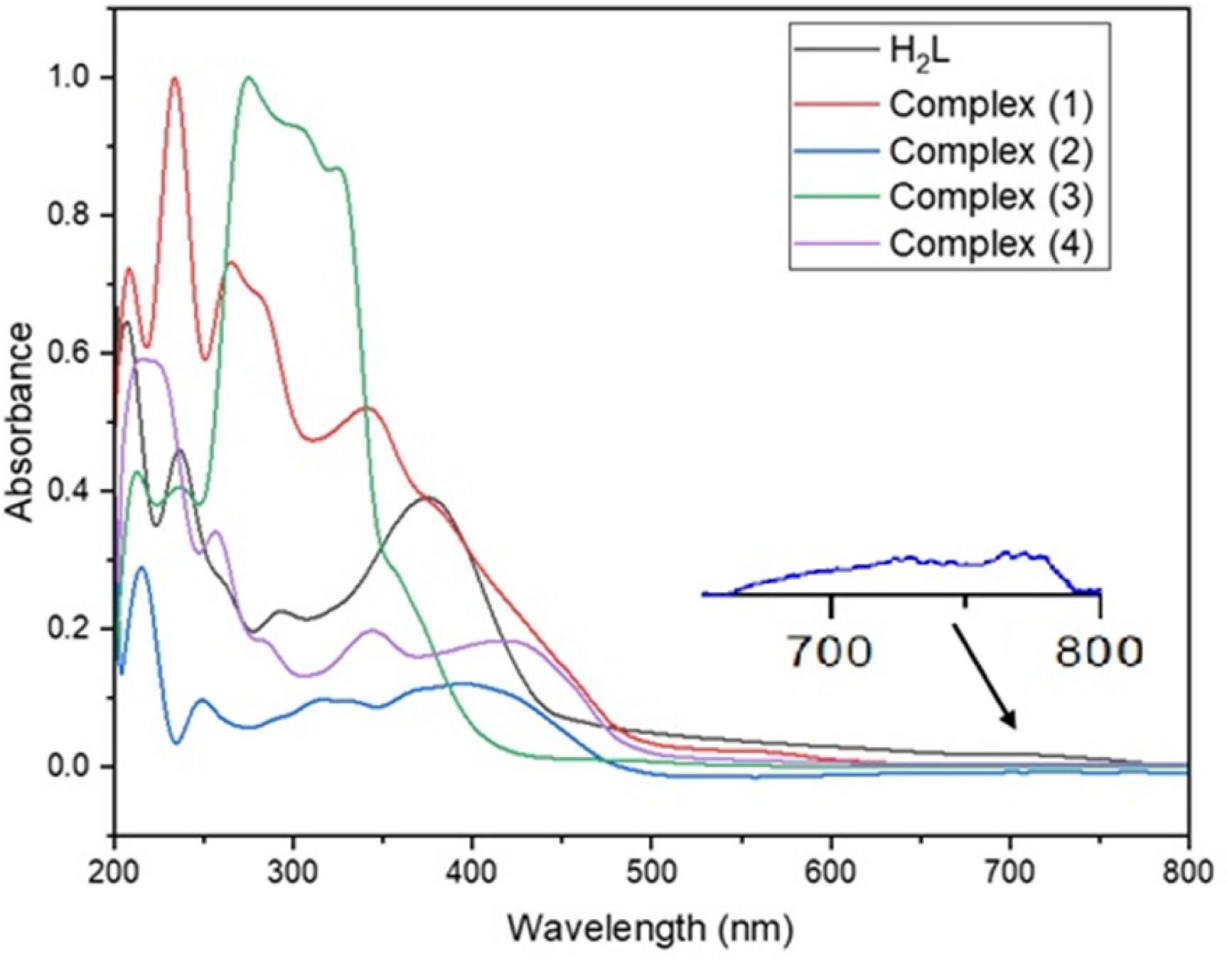
UV–Visible electronic absorption spectra of complexes **1**–**4** and used ligand **H**_**2**_**L.**

For all compounds under study dissolved in DMSO solvent, the UV–Visible spectra were performed across a broad range of wavelengths (200–800 nm) to determine the status of transitions related to ligand field, charge transfer, and intra-ligand^50^. A clear picture of the intricate geometry is provided by such transitions. Furthermore, the measurements of the magnetic moment data (μ_eff_) rigorously validated the suggested geometrical arrangement (Fig. 4, Table 3). The electronic spectra of ligand exhibited bands at 236 and 261 nm attributed to π → π* transitions in the aromatic system^[51]^. Furthermore, π → π* transition of azomethine group observed at 292 nm where n → π* of the same group observed at 376 nm^[52,53]^.

**Table 3:** Magnetic moment values and results of electronic absorption for compounds **1**–**4.**

compound	Μef (B.M.)	Transition bands (nm)	Assignment	Proposed geometry
Ligand	–	236, 261 292 376	π → π* (aromatic) π → π* (azomethine) n → π* (azomethine)	–
1	5.94	563 456	^6^A_1_ → ^4^T_1_(G) ^6^A_1_ → ^4^T_2_(G)	Tetrahedral
2	1.81	439 739	Charge transfer ^2^B_1g_ → ^2^A_1g_	Square planar
3	–	462	Charge transfer	Octahedral
4	–	425	Charge transfer	Tetrahedral

For the Mn-complex** 1**, one peak appeared at 563 nm, corresponding to ^6^A_1_ → ^4^T_1_(G)^54^. The second bans allocated at 456 nm are assigned to ^6^A_1_ → ^4^T_2_(G). The magnetic moment of such a produced compound was measured to be 5.94 BM. Both transitions and magnetic moment value confirm the tetrahedral geometry of such a complex.

The spectrum of Cu-complex** 2** showed two new peaks. The broad peak appeared at 730 nm attributed to ^2^B_1g_ → ^2^A_1g_ transition propping square planar geometrical configuration around the Cu(II) ion. The peak presented at 430 nm, is imputed to charge transfer transitions^[55]^. Cu-complex 2’s magnetic moment was defined as 1.81 BM, which favors the electronic spectrum results obtained by falling within the range of expected spin-allowed values for a single unpaired electron.

In complex **3,** the emergence of the non-ligand spectral beak at 462 nm indicated the charge transfer (CT) mode between ligand and metal ions^[56,57]^. As anticipated, this complex; Zr(IV) complex, showed no signs of paramagnetic properties or d–d electronic transitions^30^. The complex’s geometry is most likely octahedral, which is how the six coordinated system in coordinated d^0^ metals should be arranged.

No d–d electronic transitions are present in Cd-complex **4**, which has a d^10^ structure. As expected, the visible portion of the electronic spectrum of such a molecule can only have CT transition bands in its electronic spectrum^54^. Consequently, the 425 nm band that was detected identified as the cause of the CT transition. Complex **4** most likely has a tetrahedral structure.

### Thermal analysis

The thermal behaviour of the investigated nanosized metallic complexes **1**–**4** was inspected through the measurement of thermogravimetry which is regarded as a crucial resource for determining the metal chelates’ molecular structure. It offers useful details on their thermal characteristics, chemical intermediate types, leftover products from the thermal decompositions, and steps of thermal degradation. It is crucial for determining the kind and percentage of molecules of water and/or organic solvents in addition to the anionic element affixed to the complex’s metallic cores^[57]^.

The TG thermograms of the solid chelates **1–4** were interpreted in Fig. S8 and analysis of all decomposition steps through reporting the calculated and found weight loss along with the temperature ranges of decomposition are obvious in Table 4. The TG thermograms that complex **1, 3,** and** 4** decomposed through five successive decomposition steps, and complex **2** decomposed through three steps. According to the weight loss percentage analysis, the primary degradation stage. It is compatible with the loss of lattice water molecules and took place approximately between 25 and 97 °C (complexes **1** and **4**); complex **2** experienced this at 25 to 155 °C, pertaining to the coordinated water loss; and complex **3** experienced in the temperature range 25 to 110 °C, pertaining to the forfeiture of lattice and coordinated water. In the second stage, at 97–171°C, complex **1**’s coordinated water was lost. Loss of the anion chloride part occurred in the third step in complex **1** and occurred in the second step for complexes **2** and **3**. Organic part started to be lost in the third step in complex **1**, where it started in the second step in complexes **2** and **4** and in complex **3** started in the fourth stage. This degradation of the organic ligand part finally resulted in the left of the thermally stable metallic oxide as residue along with carbon ash in complexes **1** & **3**.

**Table 4:** Results of thermal analysis and their interpretation complexes 1–4.

Complex (M. Wt)	Temp. range (°C)	Mass loss		Assignments
		Calc	found	
Complex 1 [Mn(HL)Cl(H_2_O)]·H_2_O (359.69)	27–96	5.00	4.18	Loss of lattice H_2_O
	97–171	5.00	5.11	Loss of coordinated H_2_O
	171–251	14.03	13.42	Loss of lattice Cl and CH_3_
	251–442	37.57	38.12	Loss of C_6_H_3_N_2_S
	442–800	15.30	15.37	Further ligand thermal degradation leaving MnO + C residue
Complex 2 [Cu(HL)Cl(H_2_O)] (350.28)	25–155	5.13	4.56	Loss of coordinated H_2_O
	155–395	65.00	65.95	Loss of coordinated Cl + C_9_H_8_N_2_OS
	395–800	7.14	7.21	Forfeiture of rest of organic ligand leaving CuO as residue
Complex 3 [Zr(HL)Cl_3_(H_2_O)] ·0.5H_2_O (457.87)	25–110	5.89	6.22	Loss of lattice and coordinated H_2_O
	110–243	7.75	7.47	loss of 0.5Cl_2_
	243–388	7.75	7.45	loss of 0.5Cl_2_
	388–674	46.66	47.38	Loss of C_8_H_9_N_2_SOCl
	674–800	19.92	19.32	Decomposition of remaining ligand leaving ZrO_2_ + 3C residue
Complex 4 [Cd(HL)_2_]·0.5H_2_O (587.95)	25–97	1.53	1.34	Loss of lattice H_2_O
	97–283	25.03	25.08	Loss of 2Me + 2OH + C_3_HNS moiety
	283–407	20.08	20.31	Loss of C_7_H_4_NO moiety
	407–566	31.50	30.57	Loss of C_10_H_5_N_2_S moiety
	566–800	19.12	19.87	Further decomposition of ligand leaving CdO remnant

#### Thermal degradation and kinetic data

**Fig. 5:**
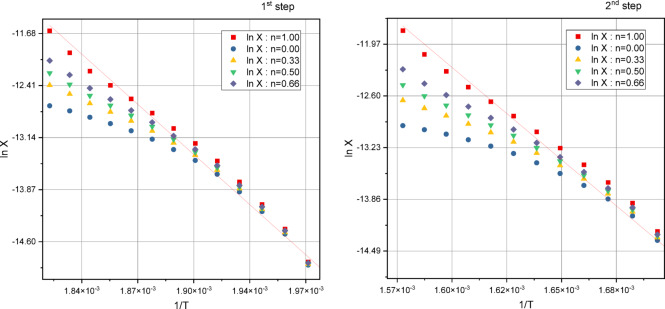
Coats-Redfern plots of 1st, and 2nd thermal decomposition steps for [Cu(HL)Cl(H_2_O)].

**Fig. 6:**
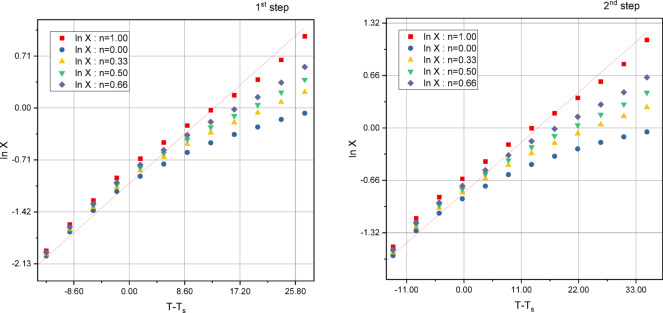
Horowitz-Metzger plots of 1st, and 2nd thermal decomposition steps for [Cu(HL)Cl(H_2_O)].

The Coats-Redfern (CR) (CR) [58]and Horowitz-Metzger (HM)^[59]^ approaches were used to assess the kinetic features of the thermal decomposition phases, as depicted in Figs. 5 and 6 for the [Cu(HL)Cl(H_2_O)] complex, with supplementary data available for additional complexes (Figs. S9–S14). The thermodynamic activation parameters were determined by applying the Eyring equation^[60]^, and the results are presented in Table S2-S5 in the supplementary information. Key observations from the data analysis (Table S2-S5, supplementary materials) include:

1. The estimated values for E, A, ∆H*, ∆S*, and ∆G* are consistently comparable across all studied complexes.
2. A first-order decomposition model (n = 1) was validated using both methods.
3. Positive ∆G* values indicate a decreasing rate of ligand removal with additional decomposition steps, reflecting increased structural rigidity in the residual complex due to ligand removal^[61]^.
4. Positive ∆S* values suggest increasing disorder as decomposition progresses^[62]^. The arrangement of activated particles is more organized than that of undecomposed components, leading to delayed degradation reactions, as indicated by negative entropy of activation (∆S*) values for certain decomposition phases in metal complexes^[60]^.
5. All decomposition processes exhibit endothermic behavior, as evidenced by positive ∆H* values.

### XRD powder technique study

X-ray diffraction technique for powder samples is one of the most used tools that can be applied to acquire microcrystalline data of the structure of compounds. It is frequently employed to provide a clear picture of the solid compounds’ crystal dynamics. The ligand (**H**_**2**_**L**) XRD patterns and its complex **1**–**4** were performed in the scattering angle 2θ range from 10 to 90°. The complexes’ diffraction pattern differs from the parent ligand’s (Fig. S15). This may be attributed to the creation of new phases because of metal ion bonding, indicating that the tested complexes were successfully made^63^. It is obvious from the pattern that the thiazole derivative (**H**_**2**_**L**) and checked metallic complexes **1**, **2** and **4** are in a good crystallinity state, whereas complex **3** was found to be relatively amorphous. This pattern of behavior for complex **3** could be explained by the relatively rare arrangement of solid frameworks during the precipitation process. When the reaction occurs quickly or the reaction mixture is cooled rapidly, the complex precipitate is frequently generated in an amorphous arrangement. In addition, It is discovered that certain chemical compounds are amorphous because their components are not able to provide a precise fit in a crystalline shape. Also, such a phenomenon could be brought on by the complicated structure’s incorporation of water or ethanol molecules. Shifted peaks in the diffraction pattern of complexes confirm the contribution of ligand groups in coordination to the metal ions. These findings are in line with the formation of metal chelates. From XRD patterns and by applying the Debye–Scherrer relationship, the 2θ and FWHM of the most intense peak are applied to determine the crystallite size (Cs) of the prepared compound in addition to their microstrain (ε) and dislocation density (D) values^64,65^ (Table 5). For the peaks located at 2θ = 17.95, 34.36, 26.99 and 35.53 nm, the Cs were calculated and found to be 34.09, 43.07, 24.95 and 43.98 nm for **H**_**2**_**L** and complexes **1**, **2** and **4**, respectively, affording nanostructures of the compounds. D values were found to be 8.60, 5.42, 16.06 and 5.16 × 10^–4^ nm^-2^, and micro-strain values are 6.812, 2.762, 6.216 and 2.775 × 10^–3^. These Nano-metric sizes of our inspected compounds are confirmed by TEM analysis, which gives us a massive expectation that these compounds can be advantageous in various fields and applications.

**Table 5:** XRD spectral data of the inspected ligand and its complexes.

Comp	2θ^a^ (º)	d^c^ (Å)	β^d^ (FWHM)	Particle size (nm)
H_2_L	12.21	7.2440	0.2479	34.09
	**17.95**	**4.9382**	**0.2466**	
	18.53	4.7837	0.2373	
	22.32	3.9807	0.3727	
	23.40	3.7991	0.3002	
	24.58	3.6184	0.2985	
	26.40	3.3731	0.2748	
	32.13	2.7836	0.4685	
1	17.48	5.07	0.298	42.95
	**34.46**	**2.6008**	**0.2024**	
2	8.30	10.6449	0.7200	24.95
	17.70	5.0056	0.6194	
	18.47	4.8000	0.6329	
	22.63	3.9262	0.4155	
	25.24	3.5250	0.5380	
	**26.99**	**3.30**	**0.3419**	
	34.57	2.5925	0.2064	
3	Not detected (amorphous)			
4	17.57	5.0449	0.5312	43.98
	**34.53**	**2.5958**	**0.1977**	

^a^θ is the scattering angle.

^b^I/I_o_ is the relative intensity.

^c^d is the interplanar spacing.

^d^β (FWHM) is the line broadening at half the maximum intensity.

### Transmittance electron microscopy study

**Fig. 7:**
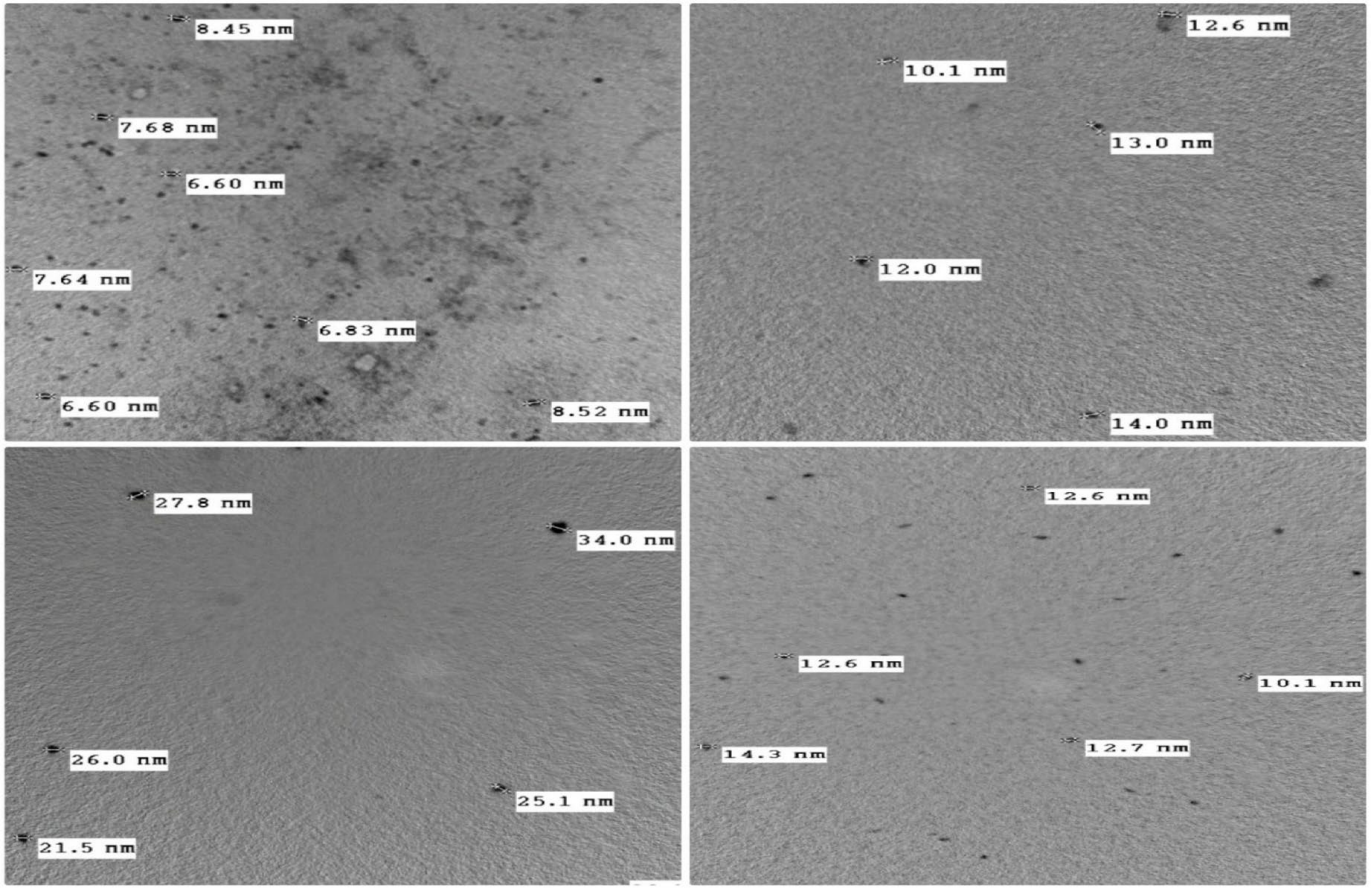
High resolution TEM images of thiazole derivative (**H**_**2**_**L**) and Cu(II), Zr(IV) and Cd(II) chelates.

Transmission electron microscopy (TEM) is a technique used to observe features such as the structure and morphology of very small specimens^66,67^. It is an alternative, beneficial quantitative approach for evaluating nanomaterials. It’s used to determine the particle size, shape and distribution with the advantage of greater resolution. For our compounds under inspection (Figs. 7 & S16) the morphology of the surface is homogeneous and consistent. In a similar vein, the images display particle consistency and proximity, suggesting the presence of a single identical material. The spherical feature often appears when the complexation sphere contains very symmetrical spherical anions. Moreover, the accumulation of several single polycrystalline particles in various ways. The photomicrograph’s dark blotches could be the result of a collection of tiny particles that have condensed. For all compounds under study, particle sizes had seemed to be in the region of nanometers which are between 6.60–8.52 nm in ligand (**H**_**2**_**L**), 31.3–31.9 nm in complex **1,** 10.1–14.0 nm in complex **2,** 21.5–34.0 nm in complex **3** and 10.1–14.3 nm for complex **4**. Compared to the bulk equivalent, these nanoscale dimensions have the potential to enhance bio-efficiency. This key trait contributes to the permeability of cell membranes of infected cells. These novel nanoparticles might be quite interesting due to their unique characteristics and possible technical applications in microelectronics, catalysis, optics, chemical sensors and biosensors^68^.

### Molecular orbital calculations

**Fig. 8:**
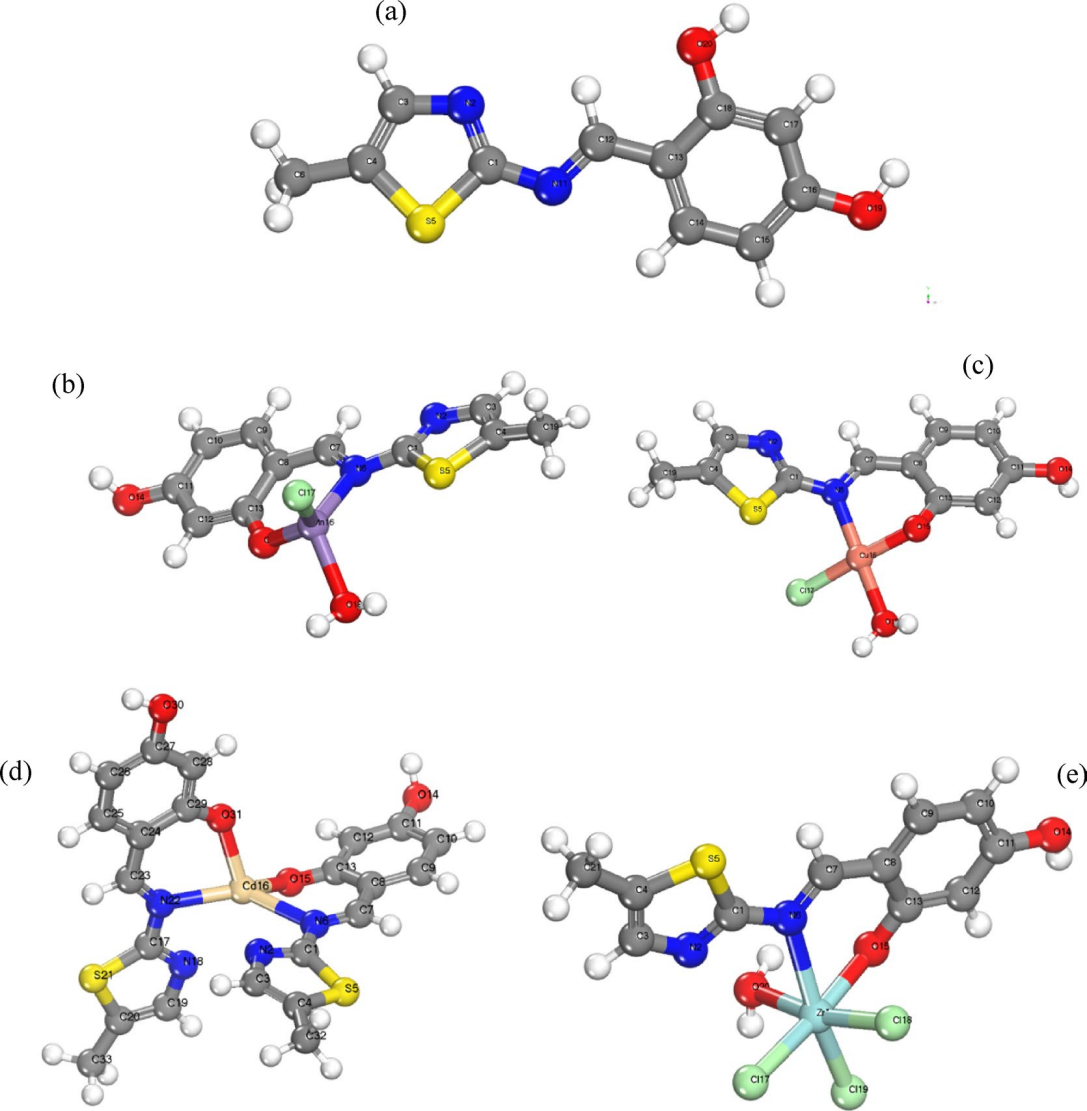
(**a**) free ligand, (**b**) Mn(II)-complex, (**c**) Cu(II)-complex, (**d**) Cd(II)-complex, and (**e**) Zr(IV)-complex optimized molecular structures.

To elucidate the molecular structures, density functional theory (DFT) quantum chemical calculations were rigorously analyzed. The bond distances and angles, detailed in Table S6 and S7 and illustrated in Fig. 8 of the optimized structural models, were comprehensively examined, leading to several key observations:

1. The C^18^-O^20^_,_ and C^12^-C^13^ bonds in the ligand (**H**_**2**_**L**) were observed to be shortened across all complexes, whereas the C^18^-O^13^_,_ and C^12^-N^11^ bonds were lengthened. These variations in ligand bond lengths were ascribed to M–O and M–N bonds that were formed with the central atoms^69^.
2. Complexation resulted in increased ligand bond angles at O^20^-C^18^-C^13^, C^18^-C^13^-C^12^, and C^13^-C^12^-N^11^ upon coordination with metal ions.
3. Interaction with metal complexes induced an elongation of the C-N^11^ bond due to establishing M–N bonds^69^, which consequently weakened the strength of the C-N group bond.
4. The M–N and M–O bond lengths were matched by the covalent radii of the M and N or O atoms put together, consistent with previously established values^70^.
5. The M-Cl bond distances in the Cu(II), Mn(II), Zr(IV) chelates are observed to be longer than the M–N and M–O bond distances.
6. The bond angles inside Mn(II) and Cd(II) complexes, were characteristic of tetrahedral complexes with sp^3^ hybridization. hybridization, while the Zr(IV) complex exhibited octahedral geometry, indicative of d^2^sp^3^ hybrid orbital configurations^[71,72]^. Lastly, the Cu(II) complex’s bond angles showed a square planner geometry with dsp2 hybridization^[60]^.

#### Chemical reactivity

The highest occupied molecular orbital (HOMO) primarily has electron-donating properties, whereas the lowest unoccupied molecular orbital (LUMO) functions as an electron acceptor. These frontier molecular orbitals (FMOs), depicted in (Fig. S17), exhibit electro-optical characteristics, electronic transition capabilities, and kinetic stability, all of which have been meticulously evaluated^[73]^. The FMO hypothesis posits the existence of a hub for coordinating electrophilic assault within aromatic compounds. Moreover, the primary mechanism driving most reactions involves the interaction between the HOMO of one species and the LUMO of another. Analytical computations reveal that the oxygen atoms in C^16^-O^19^, and C^18^-O^20^ , as well as the nitrogen atoms in C^12^-N^11^, and C^1^-N^2^, exhibit the highest molecular orbital coefficients, indicating favorable sites for complexation.

The energy gap (ΔE = EHOMO—ELUMO) and chemical properties of the separated compounds are shown in Table 6. These descriptors are derived from established equations^74,75^.

1 $${\text{Electronegativity }}\left( \chi \right) = - {1}/{2 }\left( {{\mathrm{E}}_{{{\mathrm{LUMO}}}} + {\text{ E}}_{{{\mathrm{HOMO}}}} } \right)$$

2 $${\mathrm{Potential}}\,\left( \mu \right) = - \chi = {1}/{2}\,\left( {{\mathrm{E}}_{{{\mathrm{LUMO}}}} + {\text{ E}}_{{{\mathrm{HOMO}}}} } \right)$$

3 $${\mathrm{Global}}\,{\mathrm{hardness}}\,\left( \eta \right) = {1}/{2}\,\left( {{\mathrm{E}}_{{{\mathrm{LUMO}}}} - {\text{ E}}_{{{\mathrm{HOMO}}}} } \right)$$

4 $${\mathrm{Global}}\,{\mathrm{softness}}\left( S \right) = 1/2\,\eta$$

5 $${\mathrm{Electrophilicity}}\,\left( \omega \right) = \mu^{{2}} /{2}\eta$$

**Table 6:** The determined ligand and complex quantum chemical parameters.

Compound	E_HOMO_ (eV)	E_LUMO_ (eV)	ΔE (eV)	η (eV)	σ (eV)	μ (eV)	χ (eV)	ω (eV)	S (eV^-1^)	∆N_max_
H_2_L	− 5.483	− 1.795	3.688	1.844	0.542	− 3.639	3.639	3.591	0.922	0.911
Complex 1	− 5.318	− 2.172	3.146	1.573	0.636	− 3.745	3.745	4.458	0.787	1.035
Complex 2	− 3.668	− 2.455	1.213	0.607	1.649	− 3.062	3.062	7.727	0.303	3.247
Complex 3	− 5.859	− 2.411	3.448	1.724	0.580	− 4.135	4.135	4.959	0.862	0.831
Complex 4	− 5.088	− 1.809	3.279	1.640	0.610	− 3.449	3.449	3.627	0.820	1.083

The softness  is the reciprocal of global hardness6

ΔN_max_, the maximum number of electrons that can be transferred in a chemical reaction, is computed as^76^:

7 $$\Delta {\mathrm{N}}_{{{\mathrm{max}}}} = - \mu /\eta$$

Insights from Table 6 include:

1. The stability of the isolated compounds can be verified by negative E_HOMO_ and E_LUMO_ readings^[77.78]^.
2. Because of the electron density of the coordinating atoms’ frontier molecular orbitals, the Cu(II) complex has a lower ΔE value (1.213 eV) than other compounds^[79]^.
3. A larger HOMO–LUMO gap signifies a harder, less reactive molecule, whereas a smaller gap indicates a softer, more reactive molecule. The tendency of an electron to deviate from the equilibrium configuration is represented by the chemical potential (μ), which decreases in the following order: Cu(II)-complex (-3.062 eV) > Cd(II)-complex (-3.449 eV) > ligand (-3.639 eV) > Mn(II)-complex (-3.745 eV) > Zr(IV)-complex (-4.135 eV).
4. The parameters η (hardness) and σ (softness) are indicators of molecular stability and reactivity. The ligand is a good soft Lewis base for complexation because in a complex, the metal ion functions as a soft Lewis acid^[80]^.
5. Additionally, the calculations revealed that the ligand possesses the lowest ω (3.591 eV), increasing the metal’s capacity to donate electrons^[81]^.
6. ΔN_max_ serves as an index to evaluate bond energy stability. The Cu(II) complex exhibits the highest ΔN_max_ values, surpassing those of the ligands and other complexes, confirming its superior electron acceptability.

Total energy calculations, as presented in Table 7, indicate that the binding energy of the complexes is elevated relative to the ligand, suggesting enhanced stability of the metal complexes. The Zr(IV) complex exhibits higher dipole moment values compared to the ligand, whereas the Mn(II)-complex has a lower dipole moment value than the ligand.

**Table 7:** Energetic characteristics of the complexes and ligands.

Compound	Sum of atomic energies (kcal/mol)	Kinetic energy (kcal/mol)	Electrostatic energy (kcal/mol)	Exchange–correlation energy (kcal/mol)	Spin polarization (kcal/mol)	Total energy (kcal/mol)	Binding energy (kcal/mol)	Dipole moment (Debye)
H_2_L	− 6.75 × 10^05^	− 1.49 × 10^04^	− 1.57 × 10^03^	9.69 × 10^02^	− 3.80 × 10^04^	− 7.14 × 10^05^	− 3.91 × 10^04^	2.8565
Complex 1	− 1.09 × 10^06^	− 1.81 × 10^04^	− 3.44 × 10^03^	1.16 × 10^03^	− 5.50 × 10^04^	− 1.14 × 10^06^	− 5.51 × 10^04^	1.6005
Complex 2	− 1.09 × 10^06^	− 1.86 × 10^04^	− 2.79 × 10^02^	1.11 × 10^03^	− 5.50 × 10^04^	− 1.14 × 10^06^	− 5.52 × 10^04^	1.6224
Complex 3	− 1.63 × 10^06^	− 2.34 × 10^04^	− 3.14 × 10^03^	1.17 × 10^03^	− 7.63 × 10^04^	− 1.71 × 10^06^	− 7.51 × 10^04^	5.2262
Complex 4	− 1.41 × 10^06^	− 2.85 × 10^04^	− 4.09 × 10^03^	1.96 × 10^03^	− 7.95 × 10^04^	− 1.49 × 10^06^	− 8.08 × 10^04^	3.3359

#### Molecular electrostatic potential (MEP)

The illustration provided shows an illustration showing the homogeneous electron density on the molecule surface is projected onto the electrostatic potential. This figure is critical for examining the subatomic structure and physical and chemical characteristics of the compound, such as polarity, charge distribution, and hydrogen bonding dynamics^82,83^. The interaction energy resulting from the electric charges of protons, nuclei, and electrons at a specific spatial coordinate r(x, y, z) determines the electrostatic potential, V(r)^84^.

**Fig. 9:**
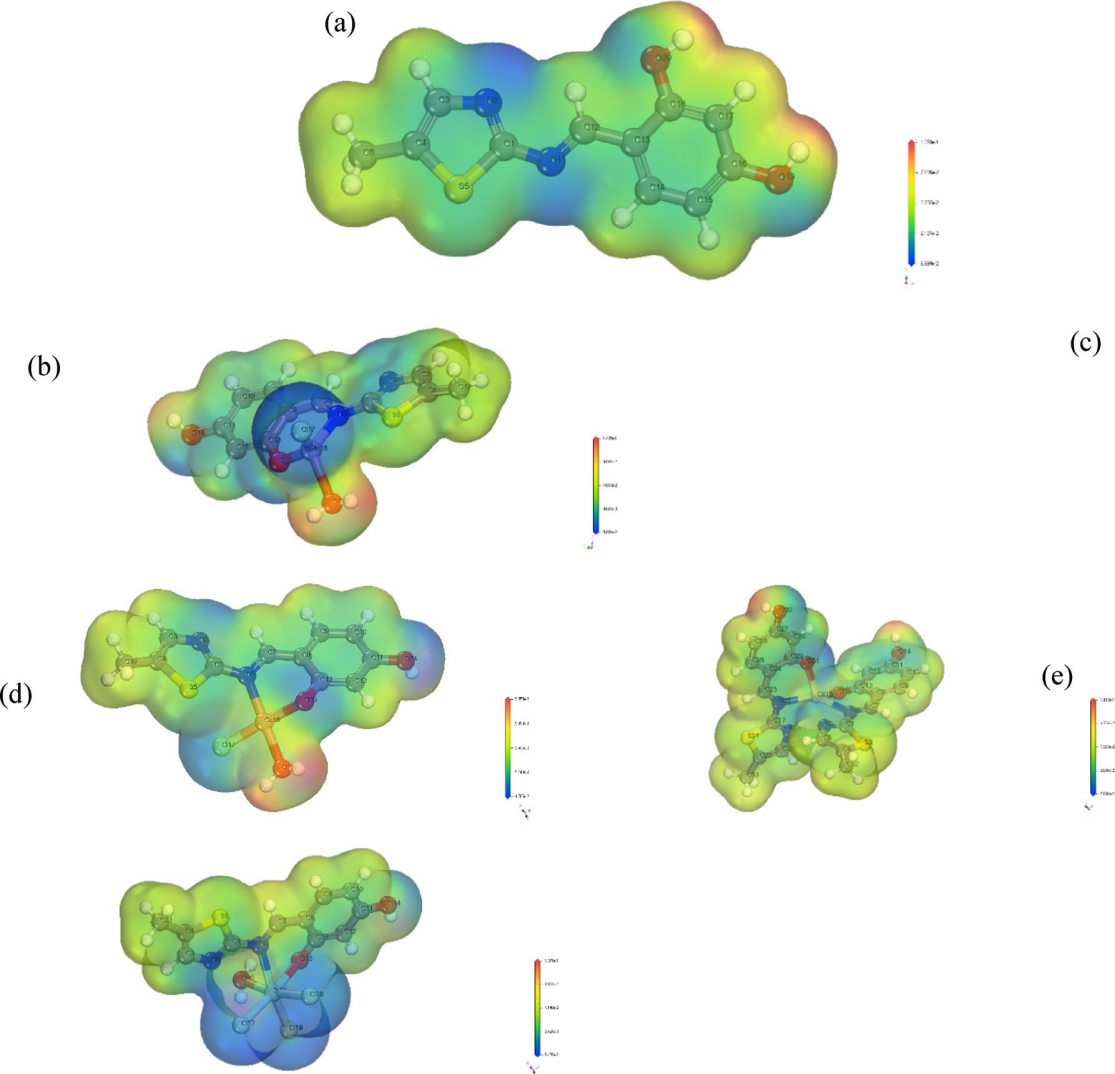
Molecular electrostatic potential maps for (**a**) **H**_**2**_**L**, (**b**) Mn(II) chelate, (**c**) Cu(II) chelate, (**d**) Cd(II) chelate, and (**e**) Zr(IV) chelate.

For atomic estimations, the Γ-point and several k-points within the Brillouin zone are used to compute electrostatic potential. In the present work, a good illustration of the molecular electrostatic potential (MEP) in three dimensions for both ligand and nanometric complexes were both generated (Fig. 9). Areas susceptible to electrophilic attack, which indicates electron acceptance, are shown in red, while regions favorable for nucleophilic attack, indicating electron donation, are marked in blue. The potential gradient is as the following arrangement: red < green < blue, with blue representing the best favorable site for attracting positive charges and red indicating the highest repulsion for negative charges. Negative potential areas correlate with negatively charged atoms like oxygen and nitrogen, whereas positive potential regions are associated with hydrogen atoms carrying a partial positive charge.

## Biological applications

### In-vitro biological study

Examination of antifungal and antibacterial biological performance of the thiazole derivatives **H**_**2**_**L** and acquired nanometric complexes towards *Bacillus subtilis* (ATCC: 6633), *Staphylococcus aureus* (ATCC: 6538) (Gram‐positive bacteria), *Escherichia coli* (ATCC:8739), *Klebsiella pneumoniae* (ATCC:13,883) (Gram‐negative bacteria), *Candida albicans* (ATCC:10,221) and *Aspergillus Niger* (ATCC:32,856) were estimated using agar diffusion technique.

The investigated compounds exhibited very significant effectiveness towards the Gram-positive bacteria; *B. subtilis*, which is clear from the value of the inhibition zone that reached 41 mm with complex **3**, which is larger than the applicable standard Gentamicin with inhibition zone diameter of 30 ± 0.1 mm (Table 8 and Figs. S18, S19). Also, with inhibition zones reaching 29 ± 0.4 mm, (for **H**_**2**_**L**) the examined compounds showed highly significant antibacterial efficacy towards S. aureus, surpassing the drug’s 24 ± 0.2 mm effectiveness. All the produced compounds demonstrated exceptionally strong antibacterial activity when tested against the Gram-negative bacterium E. coli, with inhibition zones up to 23 ± 0.2 mm, greater than the 17 ± 0.1 mm of the administered drug. A comparable pattern was shown by the materials under investigation, which showed very significant antibacterial activity against *K. pneumoniae* with inhibition zones as big as 33 ± 0.1 mm (for **H**_**2**_**L** and complex **2**), surpassing the applicable standard of 28 ± 0.2 mm. Furthermore, by testing the studied compounds against some types of fungi and by applying the standard *Amphotericin B* (35 ± 0.2 nm), which is currently in use. the ligand, complexes **2**, **3** and **4** exhibited exceptionally potent and substantial activity, having a diameter of the inhibition zone of 44 ± 0.3 mm against *C. albicans*. In case of *A. niger* fungi*,* the tested substances outperformed with good activity and has inhibitory zones of 20 ± 0.4 mm, which is almost the same as the reference drug *Amphotericin B* (22 ± 0.1 nm). This greater antimicrobial activity upon the complexation process is due to the increase in the lipophilicity character of metal chelates^67,71^. Furthermore, the improved action of the metallic compounds can be attributed to the nature of metal ions, which, compared to the unbound ligand molecule, are more hypersensitive to human cells^[85,86]^. The coordinated metal ions could interfere with the function of cellular enzymes or lead to detrimental interactions between the cell’s constituent parts^49,56^. The activity of various complexes varies among species due to either variations in the ribosomes in microbial cells or the impermeability of microorganisms’ cells^50, 87^. According to these valuable results, the studied aminothiazole ligand and nanometric complexes **1**─**4** possess an amazing deal of potential to become both novel and exceptionally effective against a broad spectrum of bacteria and fungi.

**Table 8:** The effectiveness of the thiazole Schiff base and nanometric chelates as antimicrobials **1**–**4**.

Compound	Inhibition zone diameter (mm) for six different micro− organisms					
	*B. Subtilis* (ATCC:6633)	*S. Aureus* (ATCC:6538)	*E. Coli* (ATCC:8739)	*K. Pneumoniae* (ATCC:13,883)	*C. Albicans* (ATCC:10,221)	*A. Niger* (ATCC:32,856)
H_**2**_**L**	35 ± 0.1	29 ± 0.3	23 ± 0.2	33 ± 0.1	44 ± 0.3	16 ± 0.1
Complex 1	29 ± 0.4	25 ± 0.3	18 ± 0.4	30 ± 0.2	–	12 ± 0.2
Complex 2	34 ± 0.5	18 ± 0.2	22 ± 0.1	**33 ± 0.1**	38 ± 0.5	15 ± 0.1
Complex 3	**41 ± 0.3**	18 ± 0.2	18 ± 0.3	26 ± 0.3	38 ± 0.1	15 ± 0.2
Complex 4	37 ± 0.1	19 ± 0.4	20 ± 0.2	28 ± 0.2	30 ± 0.4	**20 ± 0.4**
Gentamicin	30 ± 0.1	24 ± 0.2	17 ± 0.2	28 ± 0.2	–	–
Amphotericin B	–	–	–	–	35 ± 0.2	22 ± 0.1

#### Antitumor assessment

**Fig. 10:**
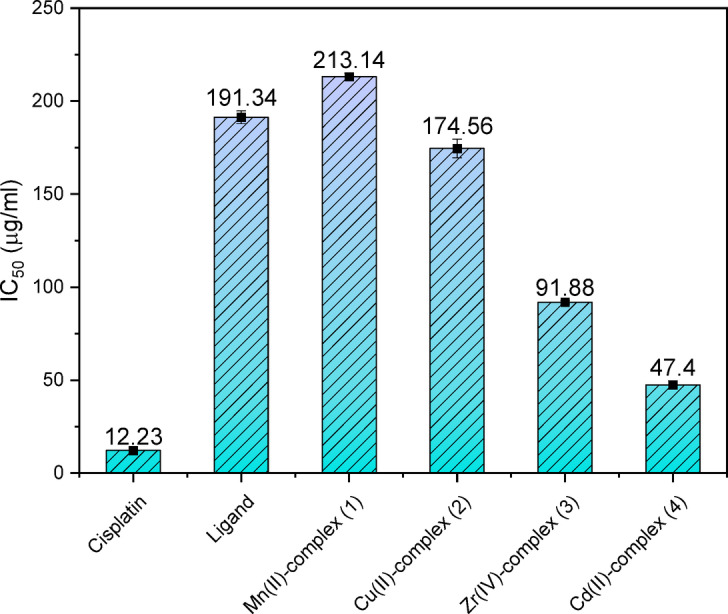
The in-vitro IC_50_ (µg/mL) of **H**_**2**_**L** and nanometric chelates **1**─**4**, contra HepG-2 cells in comparison with Cisplatin.

**Fig. 11:**
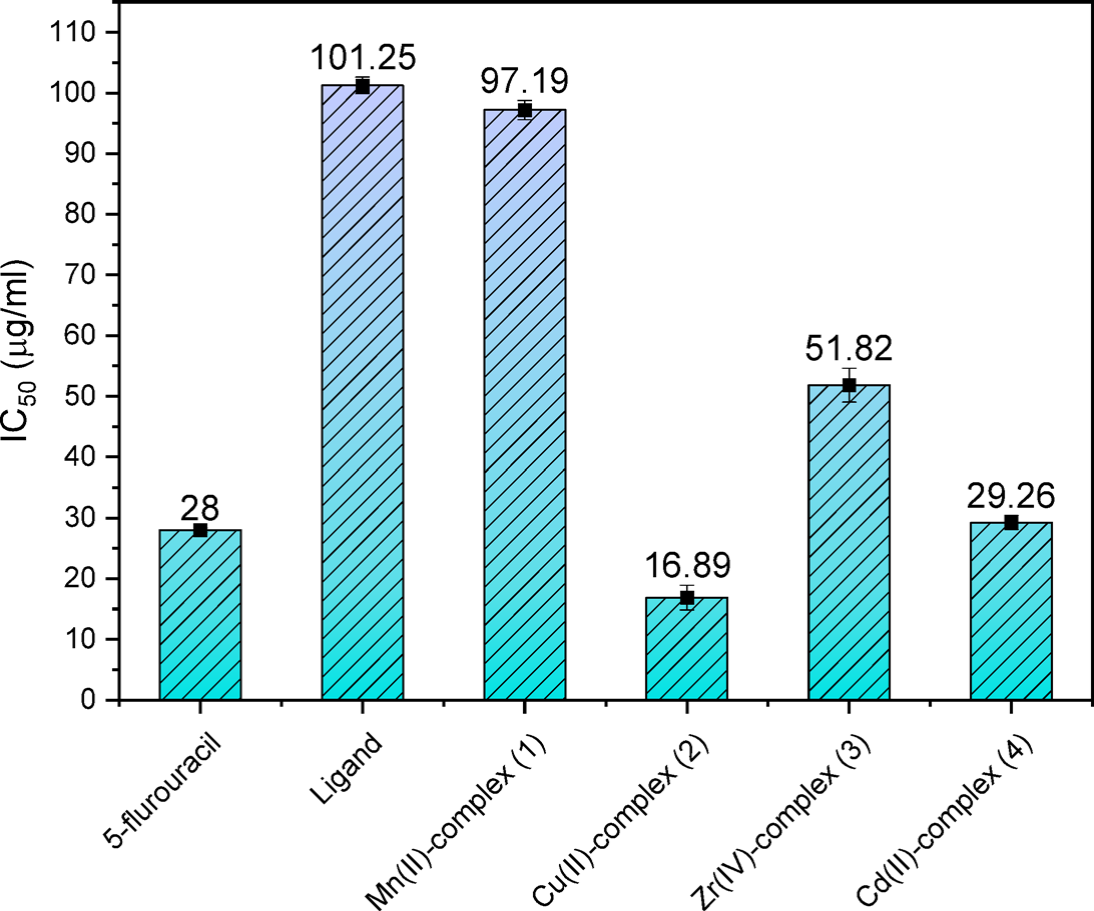
The in-vitro IC_50_ (µg/mL) of **H**_**2**_**L** and nanometric complexes **1**─**4**, against MCF-7 cells, compared with 5-flurouracil.

The primary therapy which is widely recognized against metastatic and localized tumor types is chemotherapy. Furthermore, because a sizable percentage of patients relapse after receiving therapy with proven cancer medications, the study’s findings need the creation of novel synthetic anticancer medications^[88,89]^. It is well-documented that the majority of metal-based complexes exhibit significantly lower cytotoxicity toward normal cells compared to cancer cells due to their differential redox environment and metabolic profile^90,91^. So, this study focused on explore the effectiveness of the metal complexes **1**–**4** and the free ligand (**H**_**2**_**L**) contra the human hepatocellular carcinoma cell line (HepG-2 cells) and human breast cancer (Mcf-7 cells). Since HepG-2 and Mcf-7 are the most prevalent of the numerous types of carcinomas in our surroundings, they were selected. Recently researched compounds were evaluated for their anticancer and growth inhibitory capabilities through measuring the concentration of a tested component that may limit cell growth by 50% in the same environment, the IC_50_^92^. Each data point, after being computed as the average of tri-replicates, was displayed as mean ± standard deviation. Table S8 and Figs. 10, 11 shows the IC_50_ found for all examined thiazole derivatives and nanosized complexes as well as the reference drugs. In contrast to the standard treatment of cis-DDP for hepatic cancer cells and 5-fluorouracil for breast cancer cells, Figs. S20 and S21 demonstrate the in-vitro anticancer effects of **H**_**2**_**L** and nanometric complexes **1**─**4** against HepG-2 and Mcf-7 cell lines. According to the results, the investigated compounds’ cytotoxic efficacy against HEPG-2 can be increased in the following order; complex **1** < ligand **H**_**2**_**L** < complex **2** < complex **3** < complex **4** and against Mcf-7 cells ligand **H**_**2**_**L** < complex **1** < complex **3** < complex **4** < complex **2**. These findings demonstrate how crucial ligand characteristics and metal type and characteristics are in limiting the anticancer effectiveness of metal complexes^68,93^. The found data also demonstrate that complex **4,** which gave (IC_50_ = 47.4 μg/ml) can be considered moderately active toward HEPG-2 cells in contrast to the common medication cis-DDP, which has IC_50_ value = 12.23 µg/ml. The greatest result has been achieved by complex 2, which displayed IC_50_ value = 16.89 µg/ml demonstrating that this substance works far better contra MCF-7 cells than the typical medication used (5-flurouracil) which has IC_50_ value = 28.0 µg/ml, and also the Cd(II) complex 4 gives IC_50_ value = 29.26 µg/ml is highly near to the value of the used reference. These compounds can thus be considered prospective anticancer treatment agents that could be employed as innovative drugs. The utilization of these compounds as therapeutic agents necessitates additional comprehensive and specialized research conducted by experts in the fields of medicine, pharmaceuticals, and biology.

#### Molecular docking

**Fig. 12:**
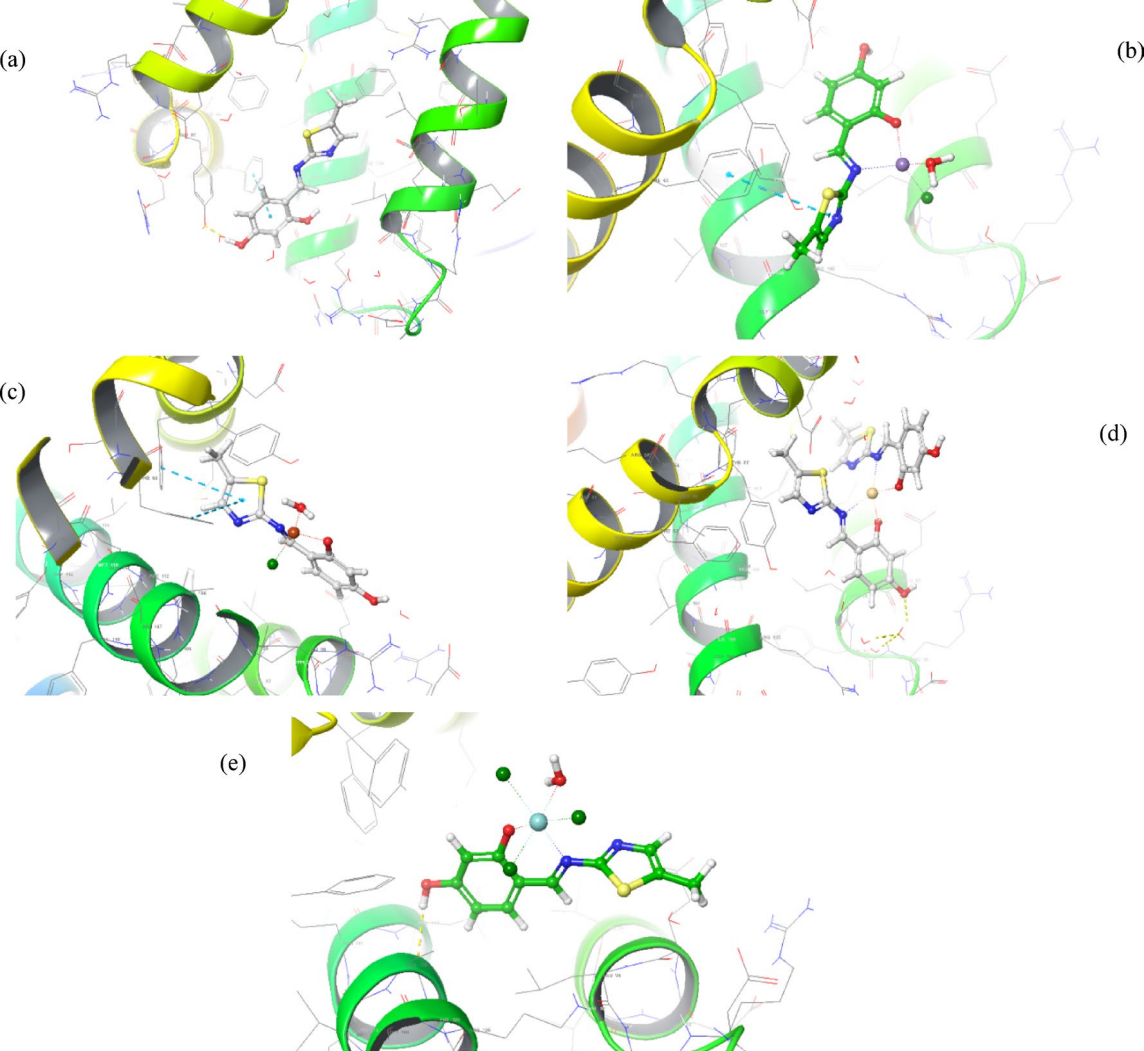
3D poses for (**a**) free ligand, (**b**) Mn(II)-chelate, (**c**) Cu(II)- chelate, (**d**) Cd(II)- chelate, and (**e**) Zr(IV)- chelate with HepG-2 (PDB ID: 2W3L).

**Fig. 13:**
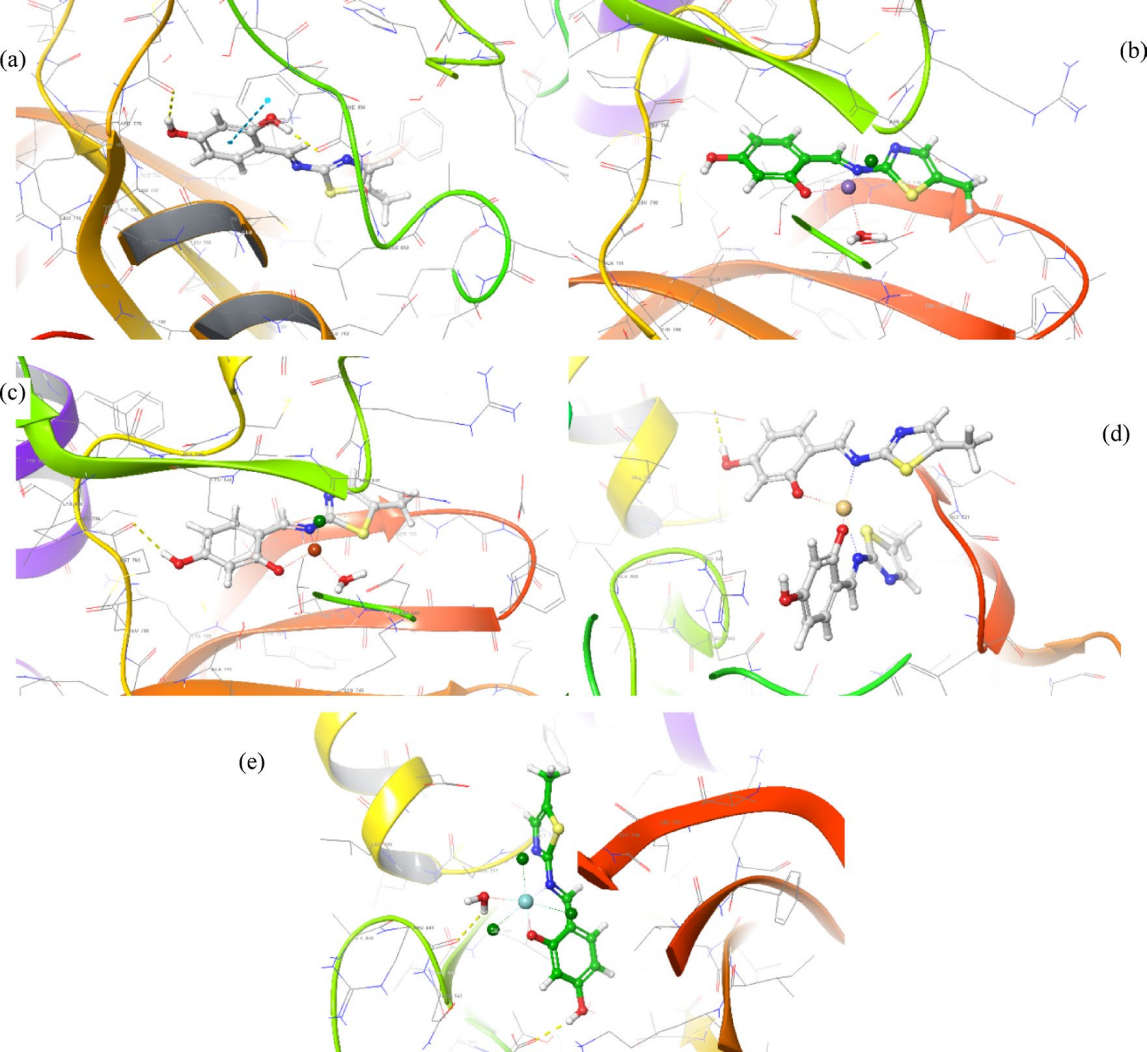
3D poses for (**a**) free ligand, (**b**) Mn(II)-chelate, (**c**) Cu(II)- chelate, (**d**) Cd(II)- chelate, and (**e**) Zr(IV)- chelate with MCF-7 (PDB ID: 3W2S).

Drug compounds’ binding affinities toward certain biological targets can be estimated using molecular docking. So, to better understand their interactions and activity against the liver cancer (HepG-2) and breast cancer cell lines (MCF-7), the thiazole-free ligand and inspected nanometric complexes were molecularly docked with the co-crystal structures of proteins for liver cancer (PDB ID: 2JW2) and breast cancer (PDB ID: 3S7S) (Figs. 12 and 13). Table 9 displays the highest Glide scores for the selected proteins and extracted compounds. Strong interactions within the generated Ligand–Protein complex are indicated by the free binding energies (G-score in kcal/mol) and the root mean square deviation (RMSD in Å) that are both noticeably negative. A quick look at Table 9 shows the following findings:

1. The ligand exhibited a robust interaction pattern with HepG-2 (PDB ID: 2W3L) proteins, with an XP G-score of -3.910 kcal/mol. Interactions included an OH → TYR67 hydrogen bond and the aromatic ring’s π-π stacking with PHE63 (distances 1.83 and 4.98 Å, respectively).
2. The Cu(II) complex showed higher interaction against MCF-7 (PDB ID: 3W2S) with an XP G-score of -5.912 kcal/mol, which may explain the high activity of the Cu(II) complex toward MCF-7 cells. The interactions of the copper ion chelate with MCF-7 (PDB ID: 3W2S) involved two hydrogen bonds [OH → MET793 and H_2_O → ARG841] with distances of 2.21 and 2.13 Å, respectively.

**Table 9:** Molecular docking scores of investigated compounds toward HepG-2 (PDB ID: 2W3L), HCT-116 (PDB ID: 1YWN) and MCF-7 (PDB ID: 3W2S) targets.

Compound	HepG-2 (PDB ID: 2W3L)				
	XP G-score (Kcal/mol)	RMSD (Å)	Interactions	Type	distance (Å)
Target 1	− 8.969	0.663	(NH_3_)^+^–– ASP70 Ar ring _o_–-_o_ PHE63 (H_2_O)∀(C = O)	salt bridge π-π stacking H-bond	3.96 5.38 2.25
H_2_L	− 3.910	2.070	Ar ring _o_–-_o_ PHE63 OH∀TYR67	π-π stacking H-bond	4.98 1.83
Complex 1	− 3.503	5.912	thiazole ring _o_–-_o_ PHE63	π-π stacking	4.99
Complex 2	− 0.469	5.211	thiazole ring _o_–-_o_ PHE63 thiazole ring _o_–-_o_ PHE71	π-π stacking π-π stacking	5.38 4.81
Complex 3	− 3.691	5.265	OH∀ALA108	H-bond	2.14
Complex 4	− 3.842	5.446	(H_2_O)∀OH	H-bond	2.21
MCF-7 (PDB ID: 3W2S)					
Target 2	− 10.912	2.035	THR790!(H_2_O)∀(C = N) THR854∀(H_2_O)∀(C = N) NH∀GLY857 OH∀ASP800 NH∀PHE856 Cl∀LEU788 LYS745∀C = O	H-bond H-bond H-bond H-bond H-bond H-bond H-bond	2.45, 2.77 2.13, 2.77 2.07 1.69 2.47 3.02 1.92
H_2_L	− 5.853	1.927	Ar ring _o_–-_o_ PHE856 OH∀PHE856 OH∀CYS775	π-π stacking Hydrogen bond Hydrogen bond	5.42 1.78 2.41
Complex 1	− 5.397	10.185	solvent exposure	–	–
Complex 2	− 5.912	10.092	OH∀MET793 H_2_O∀ARG841	H-bond H-bond	2.21 2.13
Complex 3	− 4.083	7.411	OH∀ASP855	H-bond	1.87
Complex 4	− 2.318	14.800	solvent exposure	–	–

Because of the reliance on algorithms and scoring functions and their sensitivity to the active site, the docking analysis reveals a slight disparity in practical results. Additionally, it does not account for experimental influences, microorganism characteristics, and drug transport mechanisms^94^.

#### Docking validation

For verification of the effectiveness of the docking processes, the original ligands “(1-(2-{[(3S)-3-(aminomethyl) -3,4-dihydroisoquinolin-2(1H)-yl]carbonyl}phenyl)-4- chloro-5-methyl-N,N diphenyl-1H-pyrazole-3-carboxamide inhibitor from receptor (PDB: 2W3L and 1-{3-[2-chloro-4-({5-[2-(2-hydroxyethoxy)ethyl]-5H-pyrrolo[3,2-*d*]pyrimidin-4-yl}amino)phenoxy]phenyl}-3 cyclo hexylurea inhibitor from the EGFR receptor (PDB:3W2S)” were redocked employing the identical docking strategies. The initial inhibitor of the peptide was accurately attached to the 2W3L receptor’s active site pocket by π-π stacking with PHE63, a hydrogen bond between water and the carbonyl group, as well as a salt bridge between (NH_3_)^+^ and ASP70. Conversely, the initial inhibitor connected to the 3W2S receptor’s active site pocket employing seven hydrogen bonds, including two interactions via water molecules. Re-docked original ligand was used to cover the native co-crystallized structure (Figs. S22 and S23). The re-docked inhibitor interacting with HepG-2 (PDB ID: 2W3L) and MCF-7 (PDB ID: 3W2S) receptors showed molecular docking scores of -8.969 and -10.912 kcal/mol, respectively, with RMSD values of 0.663 and 2.035 Å, respectively.

## Conclusion

In this recent study, 2-amino-5-methylthiazole and 2,4-dihydroxybenzaldehyde underwent a condensation reaction to create the aminothiazole Schiff base ligand (**H**_**2**_**L**). Mn(II), Cu(II), Zr(IV) and Cd(II) chelates were synthesized and inspected. The analytical and spectral characteristics validated the structure and compositions of the newly developed synthetic compounds. The findings collected demonstrated that the metal ions were chelated in a monobasic bidentate manner by one ligand molecule using the nitrogen of the imine (C = N) group and the ionizable *ortho*-position phenolic oxygen. UV–Visible spectra and magnetic moment confirmed formation of tetrahedral Mn(II) and Cd(II), square planar Cu(II) complex and octahedral Zr(IV) complex. XRD introduced crystalline nature of the free ligand and the Mn(II)-chelate **1**, Cu(II)-chelate **2** and Cd(II)-chelate **4**, where XRD pattern of the Zr(IV)- chelate **3** was relatively amorphous. TEM pictures of the investigated compounds exhibited a uniform and homogeneous character of the surface morphology and metal ion distribute on the complexes surfaces as nanosized particles. The molecular orbital calculations using dmol^3^ underscored the structural and reactivity changes due to complexation, highlighting stability and reactivity insights from energy gaps, chemical descriptors, and molecular electrostatic potential mapping. The newly prepared compounds exhibited high bioactivity, especially after chelation. When compared to the typical antibiotic, the antibacterial capacity of the study’s findings were very encouraging. The greatest anti-cancer activity have been achieved by Cu(II)-chelate 2 which displayed IC_50_ value = 16.89 µg/ml contra MCF-7 cells which is greater than the IC_50_ value of standard drug applied (5-flurouracil 28.0 µg/ml). Active sites docking interactions were assessed. The molecular docking exhibits significant interactions, with the ligand showing strong affinity for HepG-2 proteins and the Cu(II) complex demonstrating higher interaction with MCF-7 cell lines. These obtained findings illustrate the potential efficacy of molecular docking in predicting drug interactions with biological targets.

## Supplementary Information

Below is the link to the electronic supplementary material.

Supplementary Material 1

## Data Availability

All data generated or analyzed during this study are included in this published article [and its supplementary information files.
