# Supplementary material for: Structure elucidation and evaluation of the antimicrobial and antitumor activities of 5-methylthiazole-based Schiff base and its metal chelates
Source: Sci Rep. 2026 Mar 28;16:10738. doi: 10.1038/s41598-026-40320-0 (PMC13039548; doi:10.1038/s41598-026-40320-0)
Supplement: Supplementary file 1 — Supplementary Material 1 [file 41598_2026_40320_MOESM1_ESM.docx]

Structure elucidation and evaluation of the antimicrobial and antitumor activities of 5-methylthiazole-based Schiff base and its metal chelates

Khalid M. Wahdan ^a,^*, Hamada S. A. Mandour ^a^, Hoda A. El-Ghamry ^a,^*, Mohammed M. El-Gamil ^b^ Abdalla M. Khedr ^a,*^

^a^ *Chemistry Department, Faculty of Science, Tanta University, Tanta, Egypt*

^b^ *Department of Toxic and Narcotic Drug, Forensic Medicine, Mansoura Laboratory, Medico Legal Organization, Ministry of Justice, Egypt*

* Corresponding authors E-mail addresses: Khaled_170096pg@science.tanta.edu.eg (K.M. Wahdan), hoda.elghamri@science.tanta. edu.eg (H.A. El-Ghamry), abdallah.khader@science.tanta.edu.eg (A.M. Khedr).

Part S1: Instruments used in characterization of ligand and metal complexes

Thin layer chromatography (TLC) was used to confirm the purity of the substances being studied. The Hanna 8733 conductivity meter was used to measure the molar conductance of metal complexes in room temperature solutions of 1.00 x 10-3 M DMSO. Using a FLASH 2000 CHNS/O analyzer, elemental analysis was performed at the Regional Centre for Mycology and Biotechnology, Al-Azhar University, Egypt. Mass spectra of prepared compounds were determined by using the mass GC-2010 Shimadzu instrument at Cairo University. ^1^H-NMR spectra of the aminothiazole based ligand and its diamagnetic metal complexes were recorded using 300 MHz Varian-Oxford Mercury in DMSO-d_6_ as a solvent and the chemical shifts were recorded in ppm relative to TMS as an internal standard. An FT-IR-4100 (JASCO, Japan) spectrophotometer was used to obtain Fourier-transform infrared spectra (FT-IR) as KBr discs in the 4000-400 cm-1 range. With the use of a Shimadzu double beam UV-Vis Scanning Spectrophotometer (UV-3101 PC), the electronic absorption spectra were acquired using matched quartz cuvettes. An X-ray diffractometer (GNR, APD2000PRO, Italy) equipped with a graphite monochromator and a Cu/Kα1 radiation scanning rate of 0.03° min^−1^ was used to obtain the X-ray diffraction patterns of the materials being examined at Tanta University's Central Laboratory. The Faraday method was used to test the powdered materials' molar magnetic susceptibility. The diamagnetic corrections were calculated using Pascal's constant, and Hg[Co(SCN)_4_] was used as the calibrant. Using a TGA-50H-Shimadzu thermal analyzer, thermal measurements for nano-metric chelates were performed at room temperature up to 800 ^o^C with a heating rate of 10 ^o^C min^-1^. Transmittance electron microscopy (TEM) observations using a JEOL (Jem-1400) electron microscope (HT 200 eV; resolution 0.1432 nm; option 1.5 million) were used to analyze the morphology of metal complexes.

Part S2: Docking validation

Inhibitors were removed and re-docked to validate the docking process: 1-(2-{[(3S)-3-(aminomethyl)-3,4-dihydroisoquinolin-2(1H)-yl]carbonyl}phenyl)-4-chloro-5-methyl-N,N-diphenyl-1H-pyrazole-3-carboxamide inhibitor from receptor ‎‎(PDB: 2W3L and ‎1-{3-[2-chloro-4-({5-[2-(2-hydroxyethoxy)ethyl]-5H-pyrrolo[3,2-*d*]pyrimidin-4-‎yl}amino)phenoxy]phenyl}-3-cyclohexylurea inhibitor from the EGFR receptor ‎‎(PDB:3W2S)‎ into the effective centers applying the Schrödinger suite. Functional parameters were unchanged to ensure the inhibitors bind accurately to the active site pocket, demonstrating minimal variance from the true co-crystallized complex.


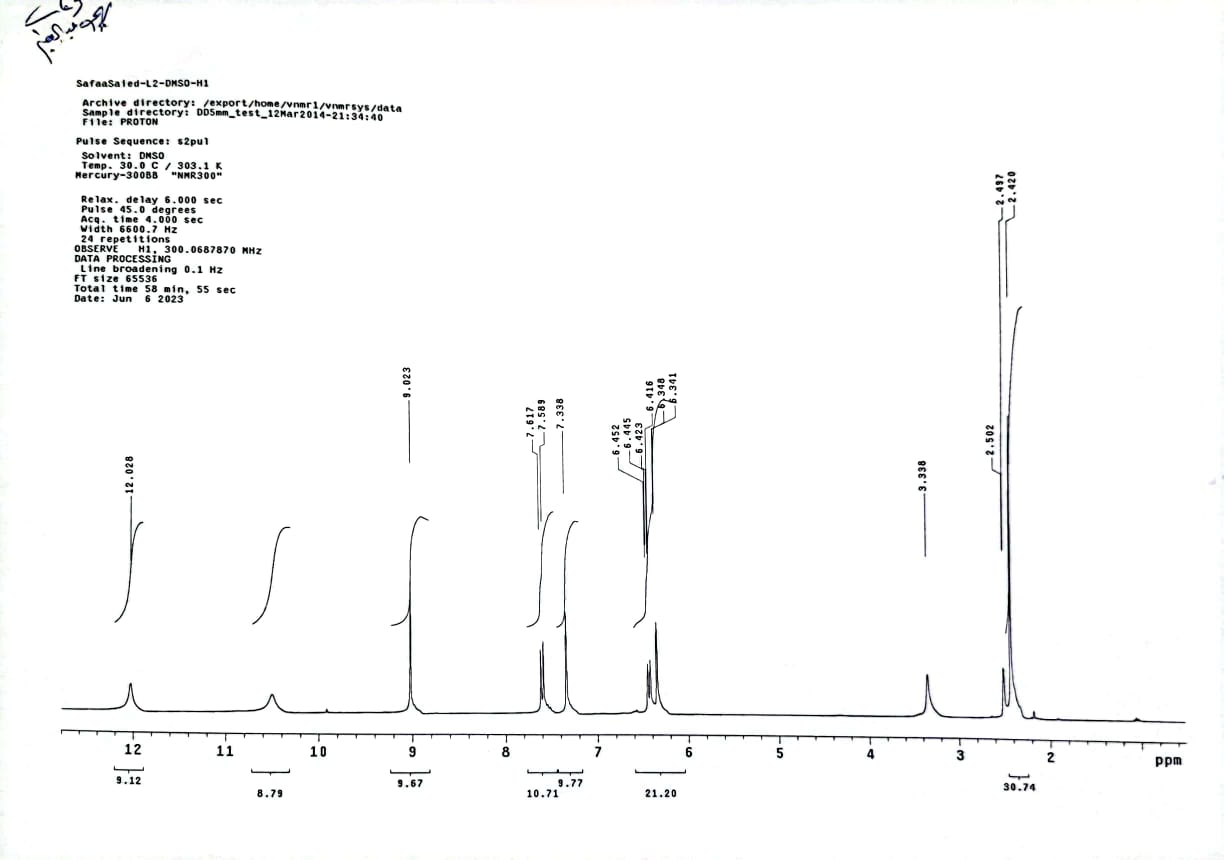


Figure S1. ^1^H NMR spectrum of the thiazole Schiff base ligand (H_2_L).


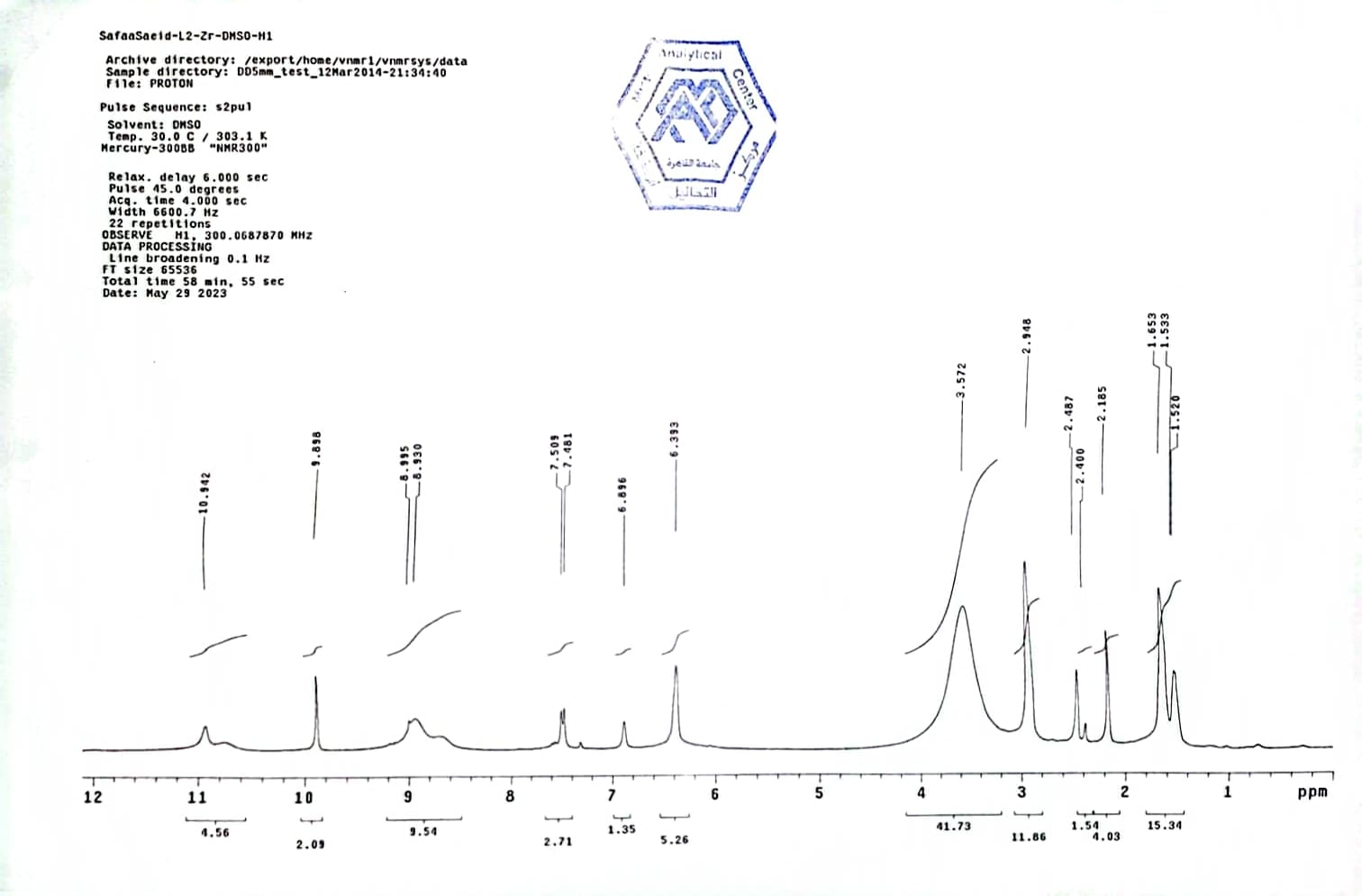


Figure S2. ^1^H NMR spectrum of Zr(IV) complex (3) with thiazole Schiff base ligand, H_2_L


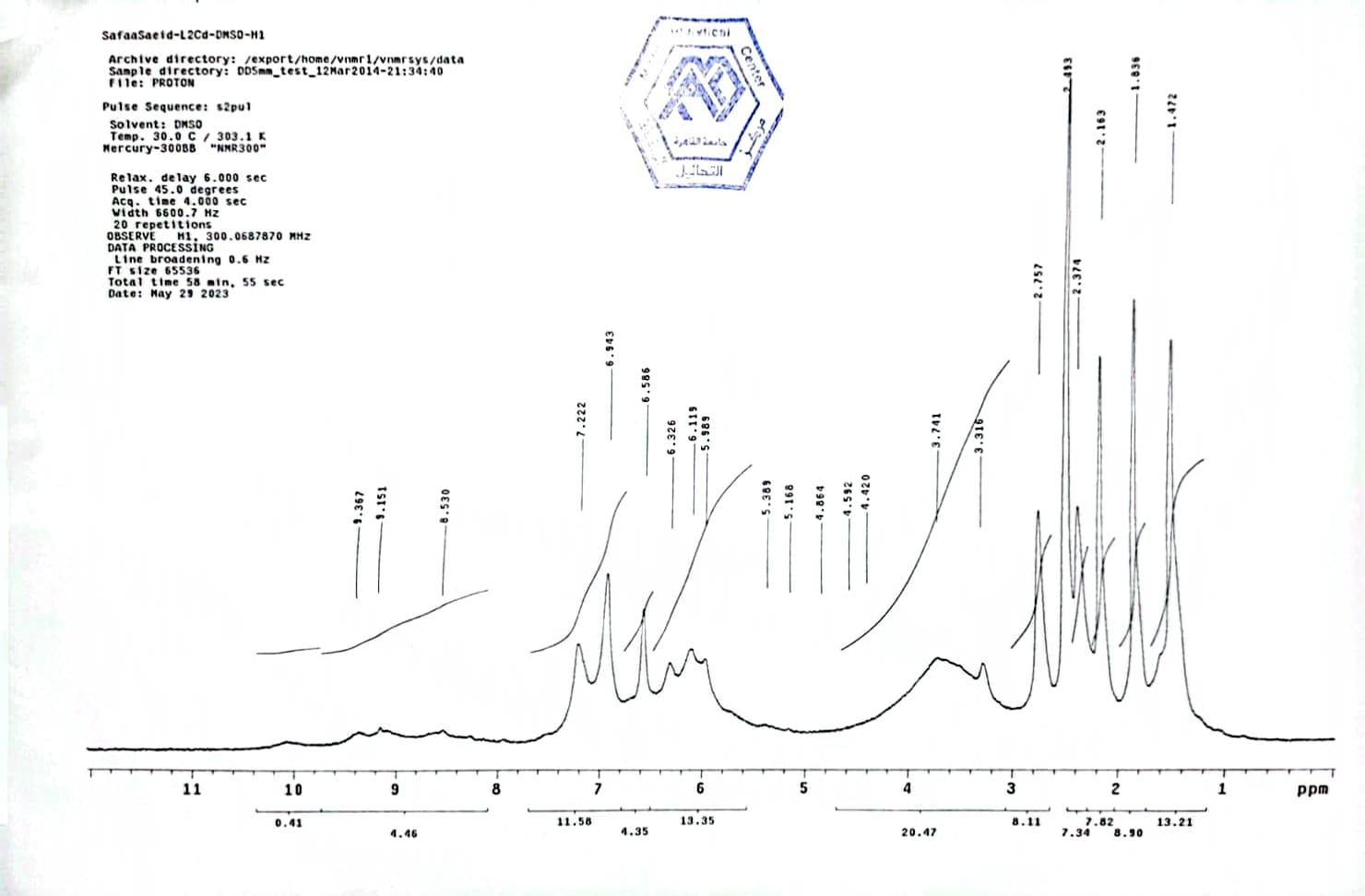


Figure S3. ^1^H NMR spectrum of Cd(II) complex (4) with thiazole Schiff base ligand, H_2_L

Figure S4. Mass spectrum of Mn(II) complex (1) with thiazole Schiff base ligand, H_2_L.

Figure S5. Mass spectrum of Cu(II) complex (2) with thiazole Schiff base ligand, H_2_L.

Figure S6. Mass spectrum of Zr(IV) complex (3) with thiazole Schiff base ligand, H_2_L.

Figure S7. Mass spectrum of Cd(II) complex (4) with thiazole Schiff base ligand, H_2_L.

Figure S8. TG thermograms of inspected nanosized metal complexes.

Figure S9. Coats-Redfern plots of 1^st^, 2^nd^, 3^rd^ 4^th^ and 5^th^ step degradation steps for [Mn(HL)Cl(H_2_O)]•H_2_O complex.

Figure S10. Coats-Redfern plots of 1^st^, 2^nd^, 3^rd^, and 4^th^ degradation steps for [Zr(HL)Cl_3_(H_2_O)]•0.5H_2_O complex

Figure S11. Coats-Redfern plots of 1^st^, 2^nd^, 3^rd^, and 4^th^ degradation steps for [Cd(HL)_2_]•0.5H_2_O complex

Figure S12. Horowitz-Metzger plots of 1^st^, 2^nd^, 3^rd^, 4^th^, and 5^th^ degradation steps for [Mn(HL)Cl(H_2_O)]•H_2_O complex

Figure S13. Horowitz-Metzger plots of 1^st^, 2^nd^, 3^rd^, and 4^th^ degradation steps [Zr(HL)Cl_3_(H_2_O)]•0.5H_2_O complex

Figure S14. Horowitz-Metzger plots of 1^st^, 2^nd^, 3^rd^, and 4^th^ degradation steps for [Cd(HL)_2_]•0.5H_2_O complex


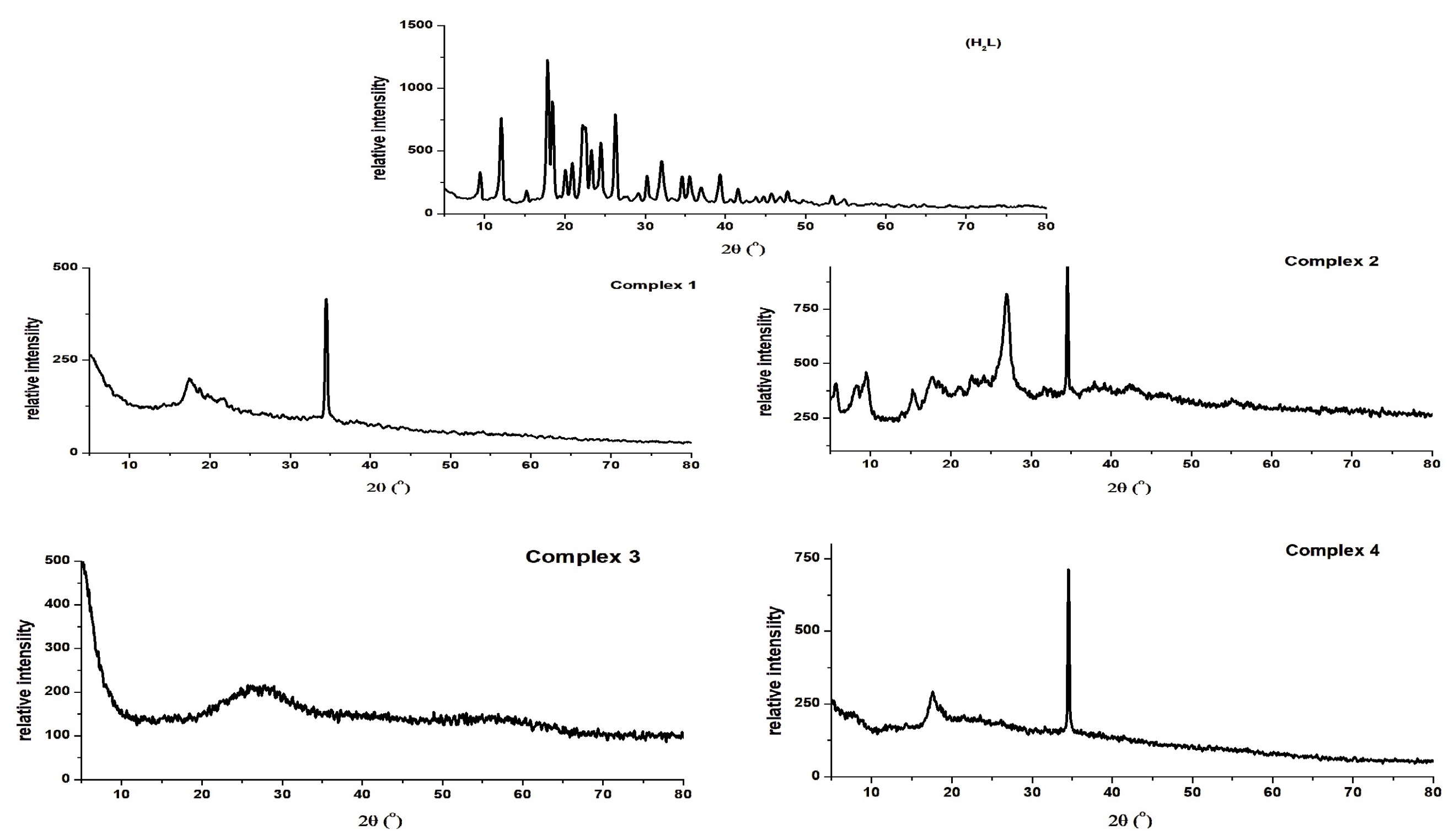


Figure S15. XRD spectral patterns of the investigated ligand H_2_L and complexes 1-4


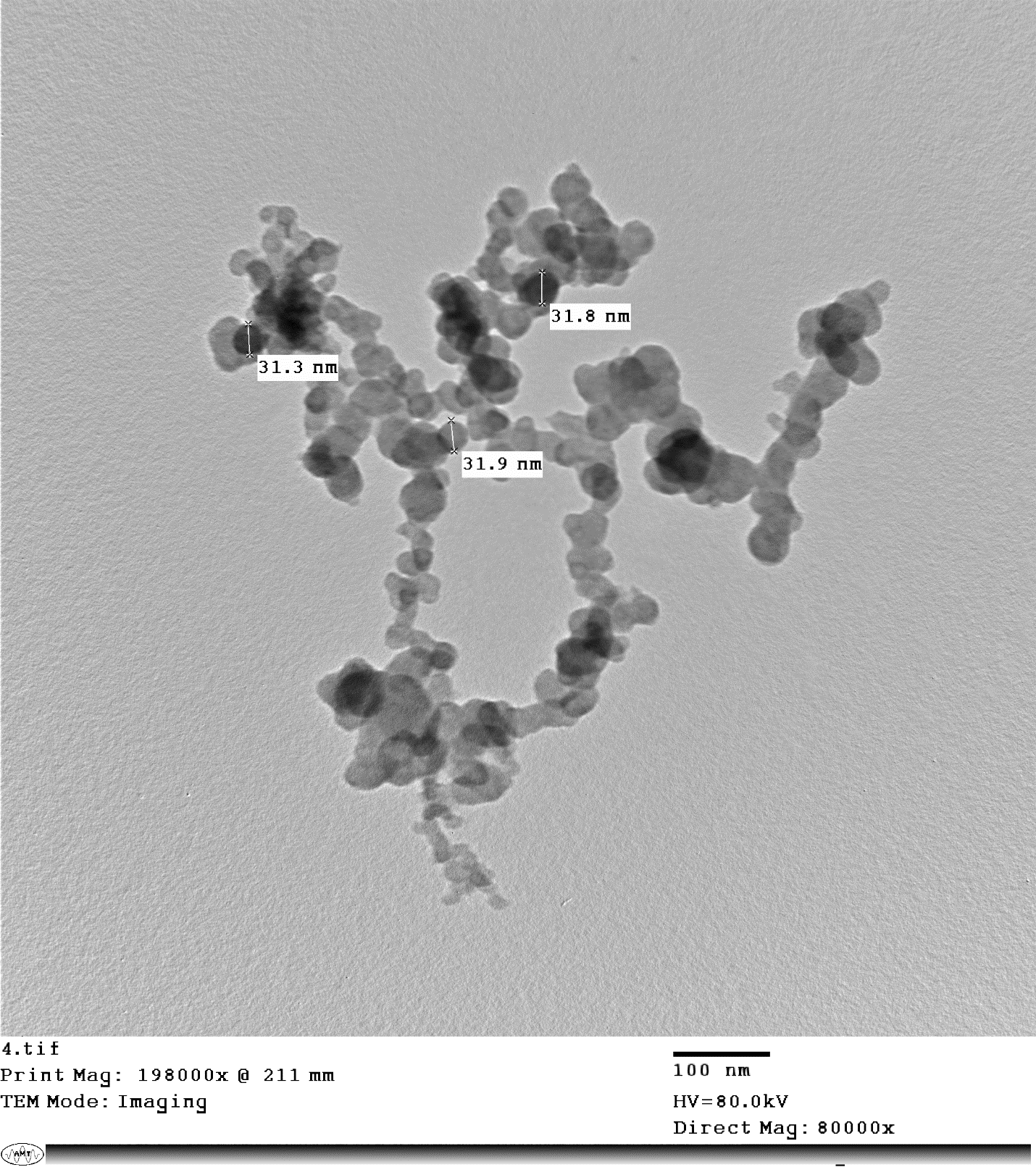


Figure S16. High resolution TEM image of Mn(II)-complexes 1

(a)


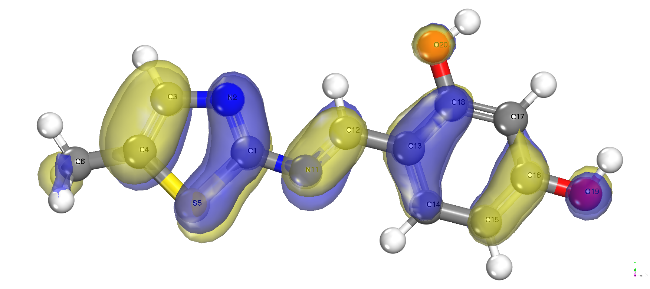

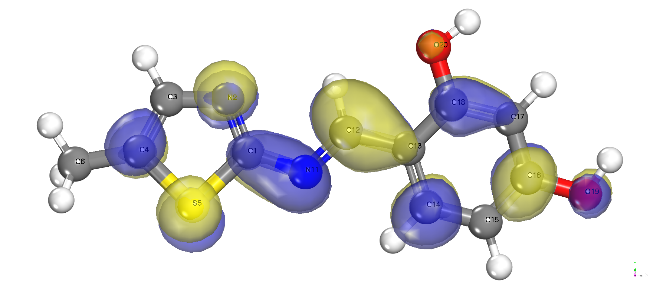


HOMO (-5.483 eV) LUMO (-1.795 eV)

(b)


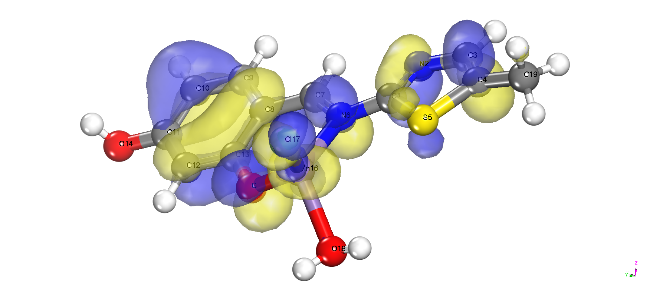

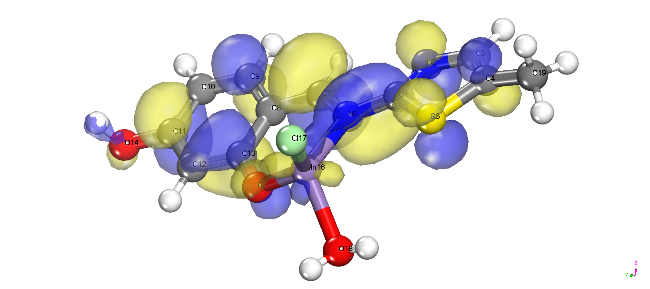


HOMO (-5.318 eV) LUMO (-2.172 eV)

(c)


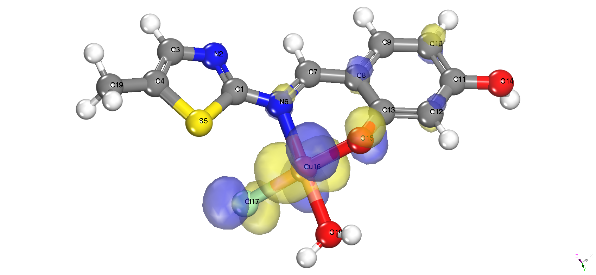

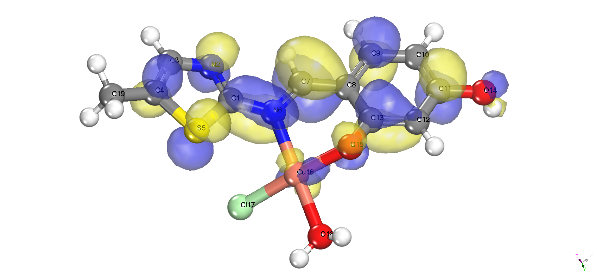


HOMO (-3.668 eV) LUMO (-2.455 eV)

(d)


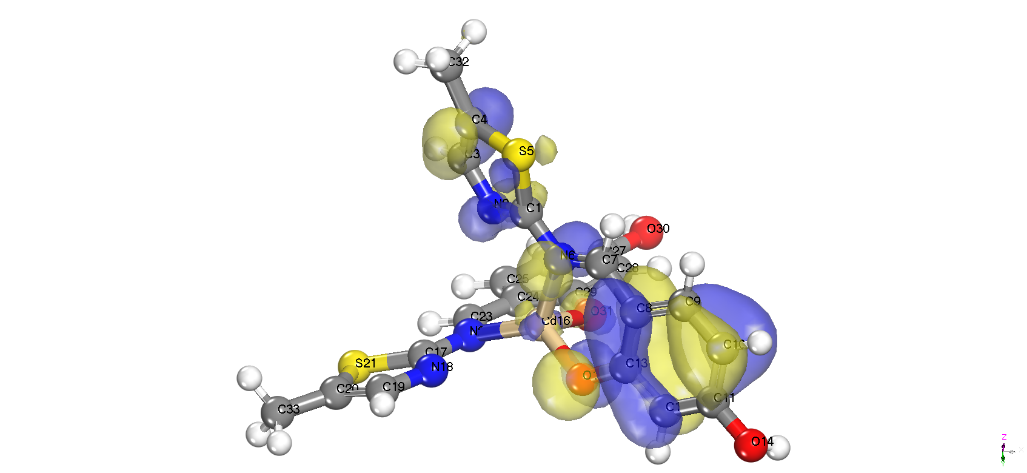

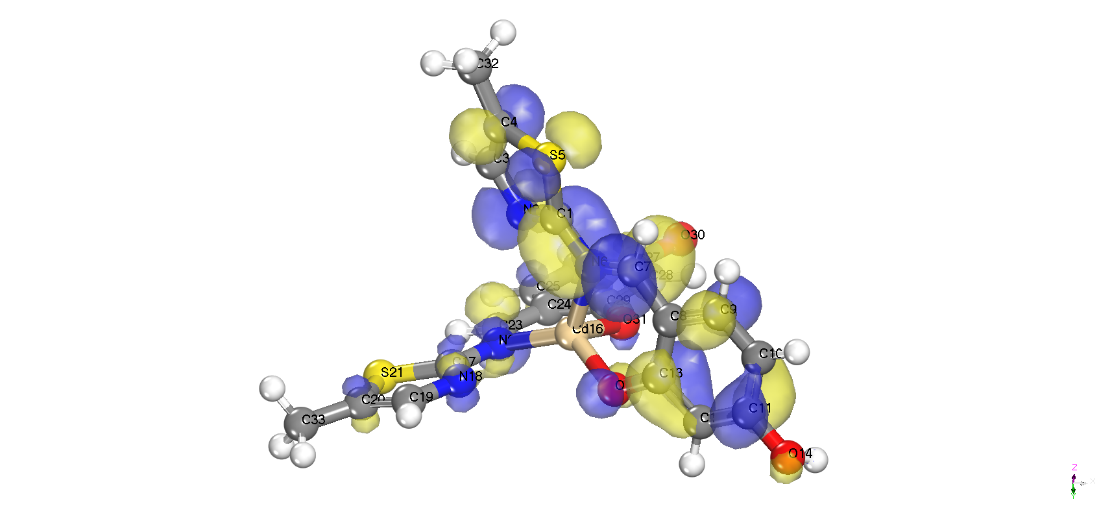


HOMO (-5.088 eV) LUMO (-1.809 eV)

(e)


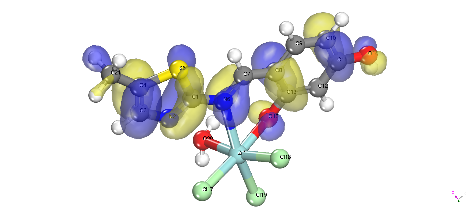

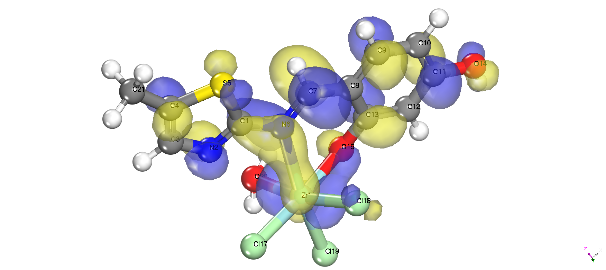


HOMO (-5.859 eV) LUMO (-2.411 eV)

**Figure S17. 3D plots frontier orbital energies using DFT method for ‎(a) free ligand, (b) Mn(II)-complex, (c) Cu(II)-complex, (d) Cd(II)-complex, and (e) Zr(IV)-complex.**


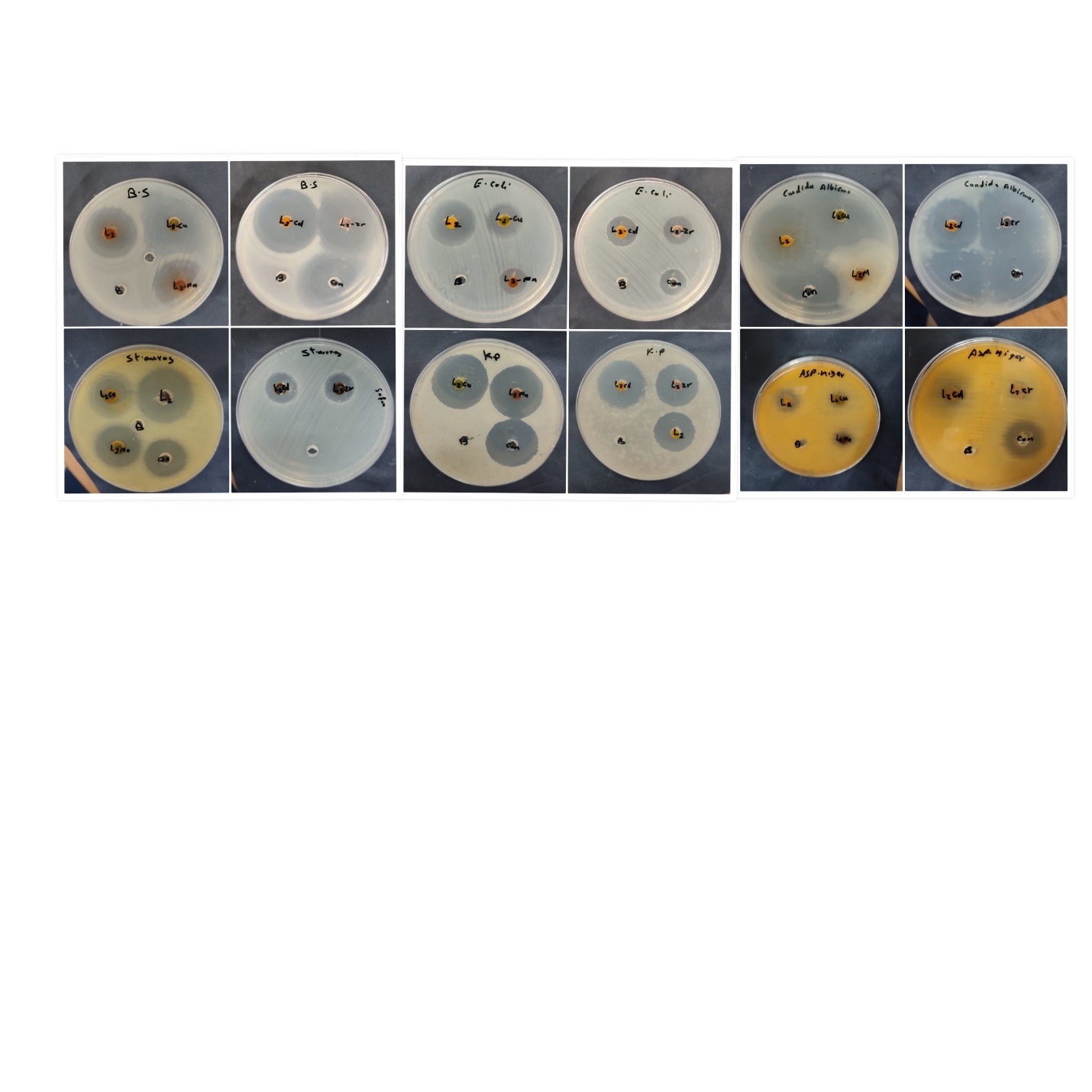


Figure S18. Plates pictures of the antibacterial and antifungal study

Figure S19. The antimicrobial activities of the thiazole Schiff base and nanometric chelates 1─4 in comparison to references standards Gentamicin and Amphotericin B.

Figure S20. The *in-vitro* antitumor activities of H_2_L and nanosized chelates 1─4 towards against HepG-2 cells compared with Cisplatin.

Figure S21. The *in-vitro* antitumor activities of H_2_L and nanosized chelates 1─4 towards MCF-7 cells, compared with 5-flurouracil (reference drug).


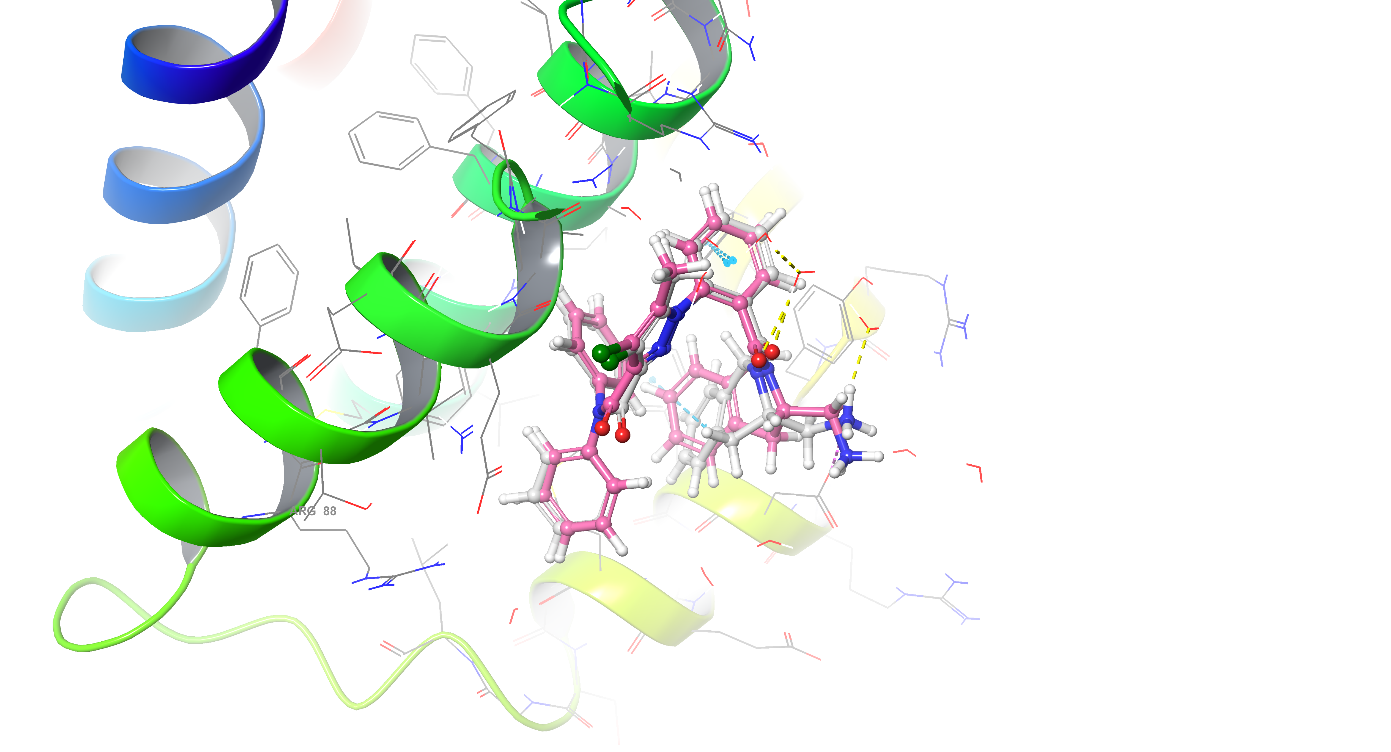


Figure S22. The overlaid of re-docked original ligand (grey) and the native co-crystallized (pink) into the receptor HepG-2 (PDB ID: 2W3L) with RMSD= 0.663 Å


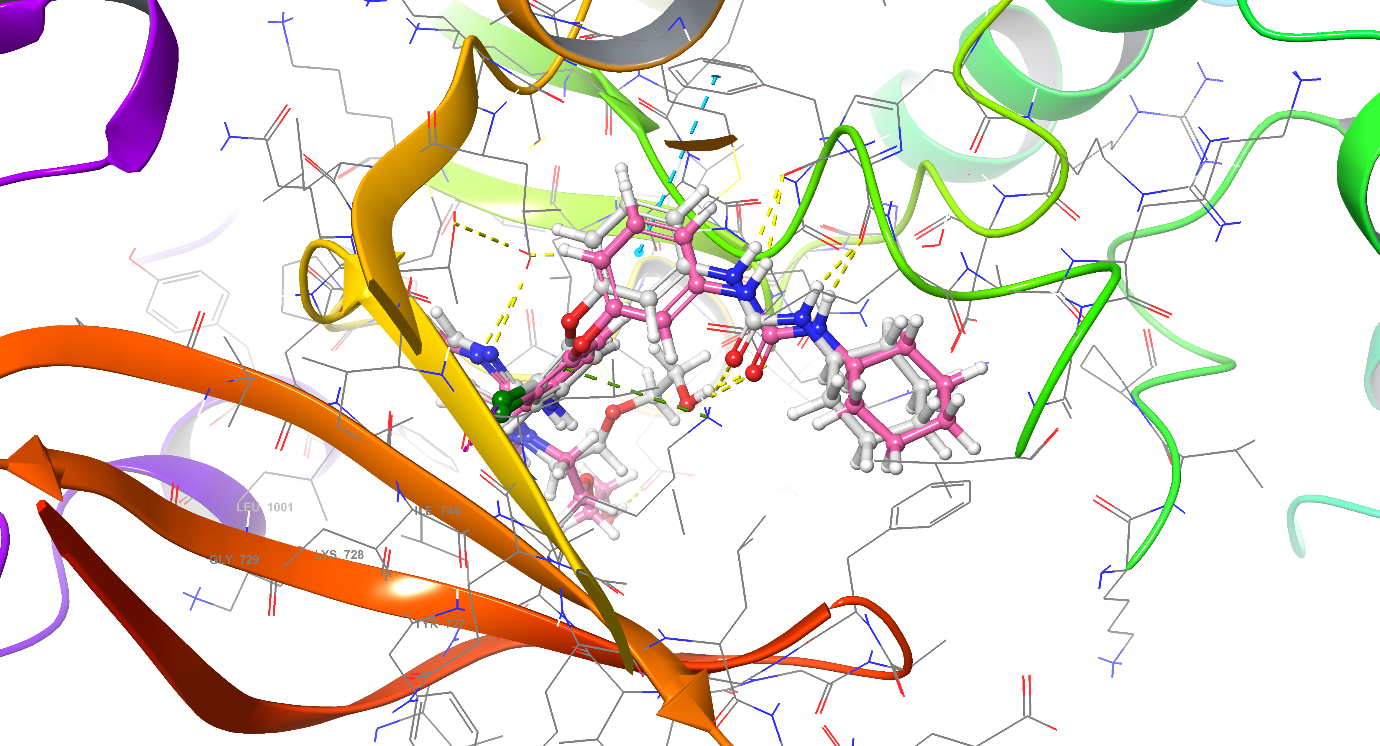


Figure S23. The overlaid of re-docked original ligand (grey) and the native co-crystallized (pink) into the receptor MCF-7 (PDB ID: 3W2S) with RMSD= 2.035 Å

Scheme S1. Suggested mass fragmentation patterns of the inspected ligand H_2_L

Scheme S2. Suggested mass fragmentation patterns of the inspected Mn(II) complex (1).

Scheme S3. Suggested mass fragmentation patterns of the inspected Cu(II) complex (2).

Scheme S4. Suggested mass fragmentation patterns of the inspected Zr(IV) complex (3).

Scheme S5. Suggested mass fragmentation patterns of the inspected Cd(II) complex (4).

Table S1. ^1^H NMR spectra obtained data

| Cpd no. | δ(O7-H) | δ(O15-H) | δ(C9-H) | δ(Aromatic protons) | -CH3 |
| --- | --- | --- | --- | --- | --- |
| Ligand | 10.5 | 9.9 | 9.023 | 6.341 - 6.452 | 2.497 |
| 3 | - | 9.898 | 8.995 | 6.393 - 6.896 | 2.487 |
| 4 | - | 9.367 | 8.53 | 5.989 - 6.586 | 2.493 |

Table S2. Kinetic parameters of [Mn(HL)Cl(H_2_O)]•H_2_O complex evaluated by Coats-Redfern (CR) and Horowitz-Metzger (HM) equations

| step | method | R^2^ | E_a_ KJ/mol | A (S^-1^) | ∆H^*^ KJ/mol | ∆S^*^ KJ/mol.K | ∆G^*^ KJ/mol |
| --- | --- | --- | --- | --- | --- | --- | --- |
| 1st step | CR | 0.9570 | 86.91 | 4.79×10^11^ | 84.16 | -0.0221 | 91.49 |
|  | HM | 0.9532 | 91.64 | 2.56×10^12^ | 88.89 | -0.0082 | 91.62 |
| 2nd step | CR | 0.9457 | 74.47 | 13.64×10^06^ | 71.06 | -0.1109 | 116.57 |
|  | HM | 0.9433 | 78.97 | 47.37×10^06^ | 75.56 | -0.1006 | 116.82 |
| 3rd step | CR | 0.9514 | 789.27 | 1.37×10^79^ | 785.01 | 1.2655 | 136.56 |
|  | HM | 0.9485 | 801.65 | 2.5×10^80^ | 797.39 | 1.2897 | 136.56 |
| 4th step | CR | 0.9782 | 1169.61 | 2.51×10^96^ | 1164.40 | 1.5943 | 166.32 |
|  | HM | 0.9779 | 1191.23 | 1.61×10^98^ | 1186.02 | 1.6289 | 166.27 |
| 5th step | CR | 0.9562 | 511.79 | 2.4×10^37^ | 506.15 | 0.4638 | 191.33 |
|  | HM | 0.9569 | 518.53 | 7.77×10^37^ | 512.89 | 0.4736 | 191.44 |

R^2^: is the coefficient of determination of order n=1.00

Table S3. Kinetic parameters of [Cu(HL)Cl(H_2_O)] complex evaluated by Coats-Redfern (CR) and Horowitz-Metzger (HM) equations

| step | method | R^2^ | E_a_ KJ/mol | A (S^-1^) | ∆H^*^ KJ/mol | ∆S^*^ KJ/mol.K | ∆G^*^ KJ/mol |
| --- | --- | --- | --- | --- | --- | --- | --- |
| 1st step | CR | 0.9475 | 172.04 | 7.28×10^14^ | 167.69 | 0.0349 | 149.41 |
|  | HM | 0.9451 | 178.08 | 2.78×10^15^ | 173.73 | 0.0461 | 149.61 |
| 2nd step | CR | 0.9647 | 166.97 | 1.41×10^12^ | 161.96 | -0.0181 | 172.87 |
|  | HM | 0.9614 | 173.09 | 4.57×10^12^ | 168.09 | -0.0084 | 173.13 |

R^2^: is the coefficient of determination of order n=1.00

Table S4. Kinetic parameters of [Zr(HL)Cl_3_(H_2_O)]•0.5H_2_O complex evaluated by Coats-Redfern (CR) and Horowitz-Metzger (HM) equations

| step | method | R^2^ | E_a_ KJ/mol | A (S^-1^) | ∆H^*^ KJ/mol | ∆S^*^ KJ/mol.K | ∆G^*^ KJ/mol |
| --- | --- | --- | --- | --- | --- | --- | --- |
| 1st step | CR | 0.9633 | 73.56 | 7.16×10^08^ | 70.65 | -0.0767 | 97.51 |
|  | HM | 0.9574 | 79.13 | 4.59×10^09^ | 76.21 | -0.0613 | 97.67 |
| 2nd step | CR | 0.9613 | 94.79 | 3.18×10^08^ | 91.03 | -0.0856 | 129.79 |
|  | HM | 0.9569 | 97.96 | 6.84×10^08^ | 94.20 | -0.0792 | 130.07 |
| 3rd step | CR | 0.9662 | 348.96 | 5.65×10^26^ | 343.77 | 0.2611 | 180.78 |
|  | HM | 0.9646 | 348.31 | 4.83×10^26^ | 343.11 | 0.2598 | 180.94 |
| 4th step | CR | 0.9622 | 157.77 | 1.67×10^07^ | 150.80 | -0.1152 | 247.35 |
|  | HM | 0.9573 | 167.21 | 6.03×10^07^ | 160.24 | -0.1046 | 247.85 |

R^2^: is the coefficient of determination of order n=1.00

Table S5. Kinetic parameters of [Cd(HL)_2_]•0.5H_2_O complex evaluated by Coats-Redfern (CR) and Horowitz-Metzger (HM) equations

| step | method | R^2^ | E_a_ KJ/mol | A (S^-1^) | ∆H^*^ KJ/mol | ∆S^*^ KJ/mol.K | ∆G^*^ KJ/mol |
| --- | --- | --- | --- | --- | --- | --- | --- |
| 1st step | CR | 0.9604 | 87.59 | 1.72×10^11^ | 84.76 | -0.0309 | 95.31 |
|  | HM | 0.9570 | 90.56 | 4.62×10^11^ | 87.72 | -0.0227 | 95.47 |
| 2nd step | CR | 0.9518 | 754.78 | 2.20×10^70^ | 750.23 | 1.0966 | 149.20 |
|  | HM | 0.9524 | 822.29 | 7.00×10^76^ | 817.74 | 1.2211 | 148.48 |
| 3rd step | CR | 0.9684 | 212.06 | 2.38×10^15^ | 206.83 | 0.0433 | 179.65 |
|  | HM | 0.9645 | 220.35 | 1.12×10^16^ | 215.13 | 0.0561 | 179.87 |
| 4th step | CR | 0.9655 | 1851.03 | 1.04×101^18^ | 1844.30 | 2.0061 | 221.11 |
|  | HM | 0.9652 | 1864.31 | 7.46×101^18^ | 1857.59 | 2.0225 | 221.15 |

R^2^: is the coefficient of determination of order n=1.00

Table S6. Selected bond lengths (Å) of the investigated ligand, and its metal complexes.‎

| Compound  Bond length (Å) | ligand | Mn(II)-complex | Cu(II)-complex | Cd(II)-complex | Zr(IV)-complex |
| --- | --- | --- | --- | --- | --- |
| C(18)-O(20) | 1.37 | 1.299 | 1.304 | 1.285, 1.289 | 1.315 |
| C(18)-C(13) | 1.405 | 1.443 | 1.443 | 1.457, 1.447 | 1.432 |
| C(12)-C(13) | 1.45 | 1.422 | 1.412 | 1.415, 1.421 | 1.414 |
| N(11)-C(12) | 1.291 | 1.326 | 1.350 | 1.31, 1.309 | 1.309 |
| M-$O_{(H_{2}O)}$ | --- | 2.259 | 2.090 | --- | 2.391 |
| M-Cl | --- | 2.3 | 2.252 | --- | 2.449, 2.368, 2.397 |
| M-O | --- | 1.981 | 1.913 | 2.196, 2.163 | 2.106 |
| M-N | --- | 2.189 | 2.063 | 2.28, 2.332 | 2.453 |

Table S7. Selected bond angles (^o^) of the investigated ligand, and its metal complexes.‎

| Compound  Bond Angle (^o^) | ligand | Mn(II)-complex | Cu(II)-complex | Cd(II)-complex | Zr(IV)-complex |
| --- | --- | --- | --- | --- | --- |
| O(20)-C(18)-C(13) | 118.106 | 123.029 | 119.722 | 124.138, 124.617 | 121.105 |
| C(18)-C(13)-C(12) | 120.37 | 124.863 | 122.741 | 124.798, 124.516 | 121.287 |
| C(13)-C(12)-N(11) | 121.662 | 127.811 | 126.552 | 126.145, 126.031 | 126.401 |
| $O_{(H_{2}O)}$-M-Cl | --- | 99.38 | 87.774 | --- | 80.932, 167.842, 99.327 |
| $O_{(H_{2}O)}$-M-O | --- | 99.823 | 92.470 | --- | 81.816 |
| $O_{(H_{2}O)}$-M-N | --- | 120.619 | 178.363 | --- | 86.935 |
| Cl-M-O | --- | 132.536 | 167.135 | --- | 92.49, 86.195, 173.393 |
| Cl-M-N | --- | 117.398 | 93.296 | --- | 161.635, 87.457, 101.454 |
| O-M-N | --- | 88.276 | 86.763 | 80.629, 80.194, 116.082, 135.126 | 72.057 |
| O-M-O | --- | --- | --- |  | --- |
| N-M-N | --- | --- | --- |  | --- |
| Cl-M-Cl | --- | --- | --- | --- | 101.695, 94.116, 92.355 |

Table S8. IC_50_ values of standard drug and compounds under study

| Compound | IC_50_ (µg/mL)  (HepG-2 cells) | IC_50_ (µg/mL)  (Mcf-7 cells) |
| --- | --- | --- |
| Standard drug | 12.23 ± 0.24 | 28.0 ± 0.37 |
| Ligand (H2L) | 191.34 ± 3.44 | 101.25 ± 1.37 |
| Mn(II) complex 1 | 213.14 ± 1.96 | 97.19 ± 1.58 |
| Cu(II) complex 2 | 174.56 ± 5.04 | 16.89 ± 2.03 |
| Zr(IV) complex 3 | 91.88 ± 0.87 | 51.82 ± 2.8 |
| Cd(II) complex 4 | 47.4 ± 2.34 | 29.26 ± 1.17 |
